# Omics Studies of Specialized Cells and Stem Cells under Microgravity Conditions

**DOI:** 10.3390/ijms251810014

**Published:** 2024-09-17

**Authors:** Fatima Abdelfattah, Herbert Schulz, Markus Wehland, Thomas J. Corydon, Jayashree Sahana, Armin Kraus, Marcus Krüger, Luis Fernando González-Torres, José Luis Cortés-Sánchez, Petra M. Wise, Ashwini Mushunuri, Ruth Hemmersbach, Christian Liemersdorf, Manfred Infanger, Daniela Grimm

**Affiliations:** 1 Department of Microgravity and Translational Regenerative Medicine, Otto von Guericke University, 39106 Magdeburg, Germany; fatima.abdelfattah@med.ovgu.de (F.A.); herbert.schulz@med.ovgu.de (H.S.); markus.wehland@med.ovgu.de (M.W.); armin.kraus@med.ovgu.de (A.K.); marcus.krueger@med.ovgu.de (M.K.); luis.gonzaleztorres@med.ovgu.de (L.F.G.-T.); jose.cortes@ovgu.de (J.L.C.-S.); petra.wise@med.ovgu.de (P.M.W.); ashwini.mushunuri@med.ovgu.de (A.M.); manfred.infanger@med.ovgu.de (M.I.); 2 Research Group “Magdeburger Arbeitsgemeinschaft für Forschung unter Raumfahrt- und Schwerelosigkeitsbedingungen” (MARS), Otto von Guericke University, 39106 Magdeburg, Germany; 3 Department of Biomedicine, Aarhus University, 8000 Aarhus, Denmark; corydon@biomed.au.dk (T.J.C.); jaysaha@biomed.au.dk (J.S.); 4 Department of Ophthalmology, Aarhus University Hospital, 8200 Aarhus, Denmark; 5 The Saban Research Institute, Children’s Hospital Los Angeles, University of Southern California, 4650 Sunset Blvd, Los Angeles, CA 90027, USA; 6 Department of Applied Aerospace Biology, Institute of Aerospace Medicine, German Aerospace Center (DLR), 51147 Cologne, Germany; ruth.hemmersbach@dlr.de (R.H.); christian.liemersdorf@dlr.de (C.L.)

**Keywords:** spaceflight, simulated microgravity, clinostat, random positioning machine (RPM), mechanobiology

## Abstract

The primary objective of omics in space with focus on the human organism is to characterize and quantify biological factors that alter structure, morphology, function, and dynamics of human cells exposed to microgravity. This review discusses exciting data regarding genomics, transcriptomics, epigenomics, metabolomics, and proteomics of human cells and individuals in space, as well as cells cultured under simulated microgravity. The NASA Twins Study significantly heightened interest in applying omics technologies and bioinformatics in space and terrestrial environments. Here, we present the available publications in this field with a focus on specialized cells and stem cells exposed to real and simulated microgravity conditions. We summarize current knowledge of the following topics: (i) omics studies on stem cells, (ii) omics studies on benign specialized different cell types of the human organism, (iii) discussing the advantages of this knowledge for space commercialization and exploration, and (iv) summarizing the emerging opportunities for translational regenerative medicine for space travelers and human patients on Earth.

## 1. Introduction

Human life in space is possible on space stations like the International Space Station (ISS) or the Chinese Space Station (CSS), in manned rockets or space crafts, and in the future, foreseen for lunar or Martian colonies. However, it is well established that microgravity, cosmic radiation, and stress adversely affect human health [1,2,3].

Space travelers face various space-associated diseases, including bone loss, muscle atrophy, cardiac atrophy, arrhythmias, renal dysfunction, immune system impairment with compromised wound healing, and neurological disorders [4,5,6,7,8,9,10].

Moreover, many alterations on the cellular level occur when cells are exposed to real (r-) or simulated (s-) microgravity (µ*g*). Human cells grown in µ*g* conditions demonstrate various morphological and molecular changes [11,12,13,14,15,16,17,18]. Human, animal, and stem cells cultured in µ*g* show modifications of the cytoskeleton [19,20,21,22], the extracellular matrix (ECM) [23,24], changes in cell adhesion [22,25], focal adhesion [25,26] and migration behavior [27,28] as well as altered cell differentiation [22,29], proliferation [22,29], survival [16], apoptosis [30], and growth [18].

The impact of r-µ*g* and s-µ*g* on the three-dimensional (3D) growth is particularly important as it facilitates the formation of organoids, multicellular spheroids (MCS), and tissues that more closely resemble the in vivo situation as a 2D cell monolayer. These 3D aggregates are formed by various cell types (e.g., chondrocytes, bone cells, thyrocytes, fibroblasts, endothelial cells, stem cells) and are used for tissue engineering, biofabrication, and bioprinting purposes [31,32,33,34]. The absence of sedimentation and convection in space creates a unique environment that enables the engineering of organoids and 3D tissues using more fluidic channels and biocompatible bioinks. These novel biofabricated aggregates are valuable for studying pathophysiological phenomena under space conditions. This new knowledge holds the potential for addressing terrestrial health issues, developing pharmacological interventions, and maintaining human health and performance during deep space exploration.

The word omics encompasses various biological disciplines, including proteomics, genomics, transcriptomics, and metabolomics. The omics field identifies, characterizes, and quantifies biomolecules, molecular processes, and signaling pathways contributing to cellular, tissue, and organismal structure and function. These levels are differentiated by their target molecules: RNA (transcriptomics), proteins (proteomics), metabolites (metabolomics), secreted proteins (secretomics), etc. 

The most rapid changes occur at the level of RNA regulation, which is both highly adaptable and transient. Protein expression regulation follows at a slower pace. Thus, proteomics typically identifies fewer alterations, while protein expression changes are often linked to meaningful functional changes. Over the past three decades, numerous research teams have demonstrated that real and simulated space conditions influence human cells’ proteome, genome, epigenome, transcriptome, and metabolome [35,36,37,38,39,40]. Omics studies in particular benefit from the integration of in-house data with external data. The NASA GeneLab is the first comprehensive omics database for space-related studies and provides researchers with data from studies primarily obtained under the influence of radiation and/or microgravity. The GeneLab includes transcriptional as well as genomic, epigenomic, proteomic, and metagenomic data from humans and various model organisms and is equipped with evaluation applications based on machine learning algorithms by the NASA AI/ML Analysis Working Group (AWG) [41,42].

This concise review focuses on publications related to omics investigations on benign specialized cells and stem cells exposed to either real space conditions or to s-µ*g* using simulation devices. The primary objective of this review is to examine omics studies of stem cells and diverse specialized cell types from the peripheral and central nervous systems, heart, blood vessels, hematological and musculoskeletal systems, thyroid, prostate, pancreas, gastrointestinal tract, and skin concerning gravity-related questions. By doing so, we aim to elucidate the underlying mechanisms contributing to the development of health problems in long-term space travelers and to generate novel ideas for tissue engineering, organoid biofabrication, and translational regenerative medicine applicable to both space and Earth.

## 2. Methods

The PubMed database was interrogated using permutations of the keywords “embryonic stem cells” or “mesenchymal stem cells” or “epidermal stem cells” or “cancer stem cells” or “ peripheral nervous system” or “central nervous system” or “eye” or “circulation” or “cardiac cells” or “cardiovascular progenitor cells” or “ vascular smooth muscle cells“ or “aortic smooth muscle cells” or “cardiomyocytes” or ”endothelial cells” or “blood cells” or “hematopoietic stem cells” or “T-cells” or “B-cells” or “killer cells” or “muscle” or “muscle cells” or “bone cells” or “bone” or “osteoblasts” or “prostate cells” or “prostate” or “thyroid” or “thyroids cells” or “thyrocytes” or “pancreas” or “gastric cells” or “intestinal cells” or “liver cells” or “hepatocytes” or “skin cells” or “keratinocytes” or “fibroblasts” or “dermal fibroblasts” or “dermal microvascular endothelial cells” with “omics”, “proteom*” or “genomic*” or “transcriptom*” or “metabolom*” or “secretom*” or “epigenom*” and “microgravity” or “weightlessness” or “spaceflight”. Studies eligible for inclusion in this review needed to analyze cultured human cells or human tissue biopsies either in real microgravity or in simulated microgravity employing ground-based devices or techniques such as the clinostat, RPM (random positioning machine), RWV (rotating wall vessel), RCCS (rotating cell culture systems), magnetic levitation, bed-rest studies, and dry immersion. This review did not include studies on malignant cells or animal cells and tissues alone.

## 3. Microgravity Platforms 

Several reviews already exist that summarize gravity-related experimental platforms. Thus, we focus on the impact of altered gravity conditions on cellular systems with respect to omics analyses. In this context, gravity-research platforms must be chosen and validated to answer each specific research question most reliably. Short-term altered gravity conditions in the second time frame might already induce responses. Here, molecular pathways that are regulated gravity dependent can be identified. Persisting changes or adaptation capacities can often only be seen after hours or even days of continuous exposure to µ*g*.

### 3.1. Methods to Alter the Impact of Gravity

For the optimal preparation of rare, expensive, and unique space experiments under r-µ*g* conditions, studies on the ground should be a prerequisite. For such kinds of studies, a portfolio of ground-based facilities aiming to simulate µ*g* conditions is available, and access can be provided by national space agencies as well as the European Space Agency (ESA) Ground-Based Facilities (GBF) program. Depending on the scientific question, several parameters must be considered, such as cell type robustness, gravity sensitivity, exposure protocols, and cultivation conditions, including, e.g., life support systems. Therefore, appropriate hardware needs to be designed, evaluated, and verified. Here, we briefly overview the most commonly ground-based simulation approaches and refer to existing reviews [43,44].

Existing µ*g* simulation devices are based on different physical principles aiming to counterbalance or randomize the influence of gravity and prevent gravity-induced factors, such as sedimentation. As gravity acts constantly and permanently, we aim to achieve a situation where the objects of interest no longer perceive gravity. Optimal simulation conditions under which the object experiences “weightlessness” depend on its sensitivity and threshold, which are unknown or at least uncertain in most cases with respect to gravity [45].

### 3.2. Simulation Approaches for Cells and Organoids

#### 3.2.1. Clinorotation, Random Positioning, Rotating Wall Vessel Exposure

**Figure 1:**
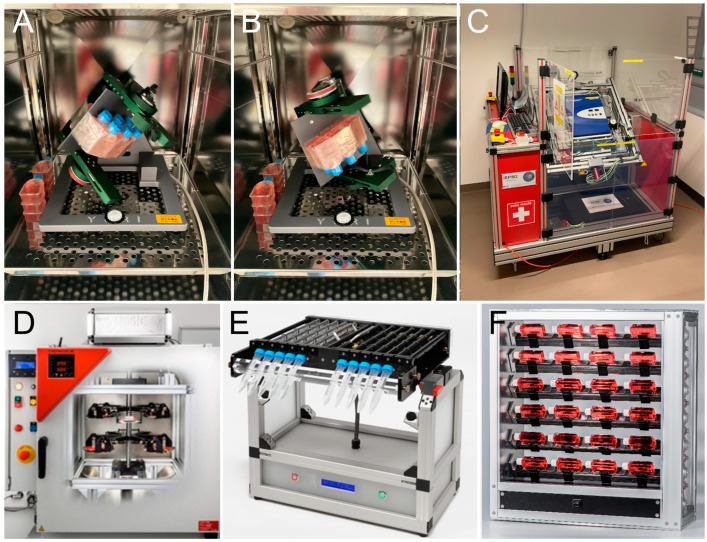
Ground-based facilities to alter the impact of gravity. (**A**,**B**) Desktop random positioning machine (RPM) placed in an incubator (Yuri GmbH, Meckenbeuren, Germany); (**C**) the random positioning incubator (RPI) developed by the “Fachhochschule Nordwestschweiz” (FHNW) and the “Eidgenössische Technische Hochschule” (ETH) Zurich, Switzerland; (**D**) MuSIC (multi-sample incubator-centrifuge) and (**E**,**F**) 2D clinostats for adherent cells or cells in suspension designed by the Institute of Aerospace Medicine, German Aerospace Center (DLR), Cologne, Germany.

Clinostats and random positioning machines (RPMs) are rotating devices characterized by their number of rotation axes and operational modes (Figure 1). A 2D clinostat consists of just one axis perpendicular to the direction of the Earth’s gravity vector. Its characteristics are continuous and constant speed and direction and, consequently, constant turning of the objects. Samples of small size are located as close as possible to the rotation axis to keep accelerations neglectable [46]. The speed of rotation has to be optimized between “too slow”, resulting in omnilateral stimulation, and “too fast”, resulting in centrifugation, to achieve a mode without polar stimulation [47,48]. 

Three-dimensional (3D) clinostats and RPMs have a second axis of rotation. While a 2D clinostat rotates with constant speed and constant direction, the two axes of an RPM rotate independently from each other and are usually set to random direction and speed. The designers postulate that the impact of gravity is averaged and approaches a minimum over time [49,50]. However, as demonstrated by biological reporter systems that are highly sensitive to shear forces, such as the dinoflagellate *Pyrocystis noctiluca*, exposure on an RPM (in its real random direction mode) induced shear stress, while fast and constant 2D clinorotation triggered neglectable shear stress responses [51].

An interesting utilization of clinorotation or random positioning is tissue engineering for cells cultivated as 3D structures. Here, morphological and gene expression changes were observed for different spheroids. The feature of constant rotation without shear forces under optimal conditions is unique for 2D clinostats that provide optimal growth environments with a high nutrient supply from the medium. This phenomenon is often employed in organoid culture conditions by utilizing shakers or rotary bioreactors to enhance nutrient supply from the medium but induces enormous shear forces on the systems that make the cultivation system rather unphysiological. The design of several types of clinostats suitable for optimal cultivation conditions for 3D cultures, spheroid, and organoid up to assemblies will need to be pushed further, as 3D cultured complex cellular networks greatly benefit from this cultivation method [52,53,54].

RWVs or RCCSs consist of a cylinder originally up to 20 cm operated under continuous, slow rotation perpendicular to the direction of the gravity vector to prevent sedimentation of the exposed samples. These techniques are usually employed for larger specimens and must be considered with the utmost care and further verification in r-µ*g* conditions [55,56,57].

Magnetic levitation: We recommend caution regarding magnetic levitation as a simulation approach for µ*g* studies on living systems. Though objects can be levitated, the influence of strong magnetic fields is immense and will trigger reactions that could cover gravity-related effects [58].

#### 3.2.2. Increased Mechanical Loading by Hypergravity Conditions

In contrast to µ*g*, opposing forces can be employed that feature increased mechanical loads, i.e., hypergravity (hyper-*g*). Here, increased gravitational loads in the physiological range of 2–10*g* are usually applied using customized centrifuges that provide hyper-*g* but with attention to neglectable shear forces, vibrations, or other environmental stimuli that might distort the measurements. The centrifuges employed for hyper-*g* studies for gravitational life science research questions range from small devices to large research platforms suitable for human-related studies. In addition to 1*g* ground controls, inflight 1*g* reference centrifuges are often used and recommended to identify the µ*g* effect from other environmental factors of spaceflight, such as radiation. Furthermore, centrifuges in space are required to determine threshold values of gravity-triggered responses.

Further, hyper-*g* conditions during experiments on drop towers, parabolic flights, or rocket launches need to be controlled for and evaluated as separate measurements from the desired phases with exposure to µ*g*. Only a comprehensive picture featuring and evaluating all acceleration phases will yield meaningful and reliable results [59].

For this reason, specialized centrifuges were designed, such as the MuSIC (multi-sample incubator-centrifuge) at DLR in Cologne, Germany, that enable exposure of various biological sample types to increased mechanical loading by hyper-*g*. At the same time, the cultures are obtained in optimal environmental conditions inside a standard cell culture incubator (i.e., controlled temperature, atmospheric conditions, and humidity) (Figure 1). In addition, swing-out gondolas and vibration-damped designs in the MuSIC provide hyper-*g* exposure of up to 50*g* without shear forces as the increased gravitational force acts perpendicular to the sample. Larger equipment, such as plate readers, electrophysiological recordings, or live-cell imaging, can be provided by the short-arm human centrifuge at DLR in Cologne, Germany. Here, various payloads can be accelerated to 6*g* and remotely controlled [60].

Studies under hyper-*g* yielded a variety of valuable results for different living systems. Live-cell imaging during 2*g* hyper-*g* has been shown to remodel key elements of astrocyte structure and decrease mobility and reactivity, which is important in astrogliosis, a defense mechanism to minimize and repair damage after brain injuries [61]. Furthermore, hyper-*g* might be a preconditioning option to avoid adaptive immune cell dysfunctions induced by s-µ*g*, as shown by T-cell activation states and cytokine secretion [62]. Hyper-*g* was used as an independent stimulus as well as a highly valuable control to decipher neuronal signaling variations under altered gravity conditions that respond by either decreased neuronal activity under hyper-*g* or increased activity during µ*g* [63].

#### 3.2.3. Simulated Microgravity Platforms for Animals and Humans

On Earth, complete weightlessness simulation for large organisms like humans and animals is unattainable. However, some of the effects observed in µ*g* can be induced by certain treatments. Gravitational unloading of weight-bearing bones and muscles can be achieved by positioning healthy subjects horizontally in a bed for a period of several days up to weeks (bed rest studies). Head-down tilt bed rest (HDT), with a 6-degree head inclination, further mimics the fluid shifts experienced in µ*g.* Countermeasures such as nutrition or centrifuge-based exercise (artificial gravity) can be combined and evaluated. Water immersion (up to the neck of the subject) as well as dry immersion (on a thin plastic sheet on a water surface) investigate the impact of a reduced hydrostatic pressure gradient on the body and induce at least some physiological functions, as in r-µ*g*. Isolation studies provide psychological and physiological data on how humans will behave during long-term social isolation and confinement stress, another aspect of long-duration spaceflight. Hind limb suspension in animal studies, such as in rats, mechanically unloads the muscle and bone system by preventing hind limb weight-bearing [64].

#### 3.2.4. Summary of Ground-Based Facilities and Recommendation

For each kind of µ*g* simulator, device-specific characteristics need to be considered, as well as potential side effects, to avoid misinterpreting the data. Each research question needs an optimal setting definition. Results should be carefully interpreted and need to be validated under r-µ*g* conditions. Furthermore, effects regarding acceleration, hyper-*g*, or other stimuli (e.g., vibration) need to be considered. However, we like to emphasize that alterations in the impact of the constant influence of gravity provide essential knowledge to prepare space experiments and increase our understanding of mechanobiology and biotechnology, such as tissue engineering and medical health issues.

### 3.3. Real Microgravity Platforms

**Figure 2:**
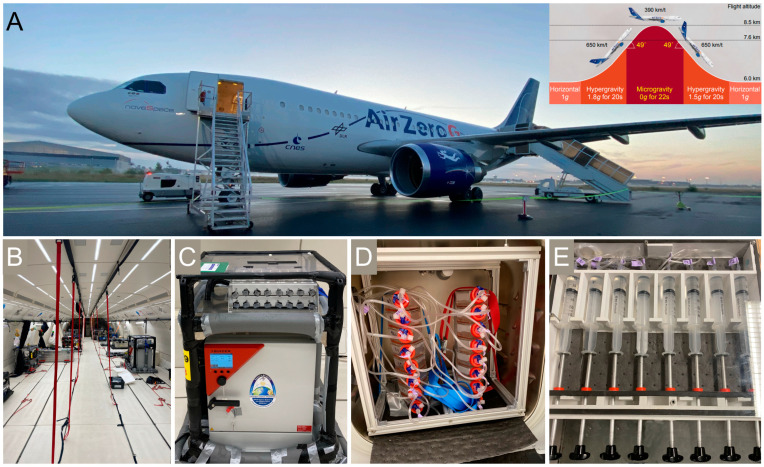
Highlights from a parabolic flight campaign (PFC). (**A**) The Airbus A310 AirZeroG aircraft operated by Novespace for the 41st DLR PFC at Bordeaux-Mérignac Airport, France. Insert shows the gravitational changes with the time sequence of a parabola; (**B**) inside of the AirZeroG aircraft allowing installation of several in-flight-operable experimental racks; (**C**) example of a PFC flight rack with an incubator and injection unit; (**D**) inner part of the incubator showing installation of tissue culture flasks harboring living human cells. Each flask is connected to the injection unit by a separate tubing; (**E**) the injection unit is used to fix the cells, as shown during maintenance and experimental setup.

Experiments in r-µ*g* are the gold standard research in this field, and access to microgravity environments is typically provided through opportunities announced by national agencies or organizations such as the European Space Agency (ESA) and the National Aeronautics and Space Administration (NASA). Proposals are reviewed based on scientific and technical merit and feasibility and are then adapted to the operational requirements of the µ*g* platform. Access is provided to, e.g., the ZARM Drop Tower, which provides up to 10 s of µ*g* [65], to parabolic flight maneuvers of airplanes like the Airbus A310 ZERO-G, providing repeated periods of reduced gravity up to 20 s of µ*g* or Moon or Martian *g*-levels accompanied by periods of hyper-*g* (about 1.8*g*). Here, the experimenter must carefully select the appropriate phase of the parabola or overall acceleration profile to address their research question [66] (Figure 2). Uncrewed sounding rockets are utilized for scientific research, providing 3 to 13 min of µ*g*. The big advantages are minimum safety constraints, late access up to 1 h before launch for experiment installation, and early retrieval of the sample after the flight, typically after 1 h. Many flight-proven experiment modules for different scientific questions exist. They are, for example, designed for optimal cell cultivation (temperature, pressure, etc.), online electrophysiological measurements, and chemical fixation of adherent cells, suspension cultures, or organoid samples at dedicated time frames [67]. Reusable modules and hardware containers are developed using conventional and advanced modeling techniques and biocompatible 3D printing technologies. Recently, the successfully launched DLR rocket campaign MAPHEUS-14 contained several biological experiments that were performed in parallel. Online electrophysiological measurements of neuronal networks on a multi-electrode array (MEA) system were recorded. In-flight chemical fixation of biological samples during various acceleration phases was performed on human IPSC-derived motoneurons and primary murine astrocytes for subsequent proteomic profiling. Further, inflight chemical fixation on human IPSC-derived brain organoids for transcriptomic analyses was conducted. This demonstrates the experimental capacities for multi-omics profiling during even a single rocket flight. Prolonged periods of µ*g* are provided by new space initiatives and the ISS. As a well-established crewed Earth-orbiting platform, the ISS allows long-term scientific research in the unique space environment. Control experiments with respect to the launch and landing acceleration scenario and radiation must be considered. We must keep in mind that the ISS orbits the Earth at an altitude of around 400 km, and thus, it is not protected by the Earth’s atmosphere. Consequently, radiation levels around the ISS increase by a factor of about 30, which must be discussed in the context of experimental data.

## 4. Results for Omics Studies in Stem Cells and Specialized Cells Exposed to Microgravity

### 4.1. Stem Cells 

Stem cells can self-replicate and differentiate into specialized cells and are regarded as promising resources for regenerative medicine and tissue engineering. Recent studies have demonstrated that r- and s-µ*g* conditions profoundly influence stem cell behavior, primarily enhancing self-renewal and maintaining pluripotency while reducing differentiation susceptibility.

#### 4.1.1. Embryonic Stem Cells (ESCs)

Controversary results were observed for ESC proliferation, with some studies [68] indicating no significant impact of s-µ*g* (3D clinostat) on proliferation from proliferating cell nuclear antigen (PCNA) expression. In contrast, others [69] suggested enhanced proliferation and survival of ESCs from data in space and thus r-µ*g* conditions. In addition, s-µ*g* decreased adhesion rates and increased apoptosis rates in ESCs [68]. Other studies demonstrated that undifferentiated mESCs exposed to alternate hyper-g and r-µ*g* phases expressed several genes associated with developmental/differentiation and cell cycle processes, suggesting a transition from the undifferentiated pluripotent to a more differentiated stage of mESCs [70]. Another study reported an enhanced induction of definitive endoderm differentiation of mouse embryonic stem cells exposed to s-µ*g* (rotary bioreactor) [71]. In addition, a further investigation focused on the effects of spaceflight on the ability of mESCs to differentiate and generate the cell lineages present in terminally differentiated tissues as a model for adult stem cell-based tissue regeneration. r-µ*g* exposure inhibited the ability of EBs to differentiate and express terminal differentiation markers for most lineages of the three primary germ layers, including bone, muscle, immune system, renal system, liver, lung, and pancreas [72]. There is evidence that spaceflight exposure of cells and tissues inhibits the proliferation and differentiation of stem cells, resulting in decreased stem cell-based tissue regenerative potential [72].

#### 4.1.2. Mesenchymal Stem Cells (MSCs)

Liu et al. [73] assessed various parameters related to oxidative phosphorylation (OXPHOS) during osteogenesis of MSCs under s-µ*g* (2D clinostat) conditions, including the level of peroxisome proliferator-activated receptor γ coactivator 1α (PGC-1α), mitochondrial DNA (mtDNA) copy number, mitochondrial mass, and oxygen consumption rate. s-µ*g* was found to inhibit both osteogenic differentiation and OXPHOS in MSCs, and the expression of sirtuin 1 (Sirt1), an important energy sensor, significantly decreased under s-µ*g* conditions. The findings suggested that targeting OXPHOS, possibly through interventions that modulate Sirt1 expression, could be a promising approach for mitigating bone loss in µ*g* conditions. Lv et al. [74] found that s-µ*g* (2D clinostat) promoted YAP expression and nuclear translocation in MSCs. Verteporfin (VP), an inhibitor of YAP, restored s-µ*g*-induced mitochondrial dysfunction and senescence in MSCs by inhibiting YAP expression and nuclear localization. 

The study of Pala et al. [75] showed that Wharton’s jelly mesenchymal stem cells (WJ-MSCs) exhibited a rapid adaptive response when exposed to an RPM. Within the first 6 h of RPM exposure, WJ-MSCs showed overexpression of the *Oct-4* gene, crucial for maintaining stemness and promoting glycolysis as a metabolic adaptation to counteract senescence. After 12, 24, and 48 h, the expressions of *Oct-4*, *SOX2*, and *NANOG* were significantly downregulated, indicating a reduction in stemness and an inclination towards cellular senescence. Genes involved in cell cycle arrest and senescence, such as *p16*, *p19*, *p21*, and *p53*, were initially upregulated, reflecting a stress-induced response. Over time, *p16* remained elevated while *p19*, *p21*, and *p53* were downregulated, suggesting that s-µ*g* induced a state of mild senescence, indicating a balance between initial protective responses and subsequent adaptation leading to senescence. Both pro-apoptotic (*BAX*) and anti-apoptotic (*BCL2*) genes were upregulated initially, followed by a decrease after extended RPM exposure, suggesting a complex interplay between survival and apoptosis. Heat shock proteins like *HSP60* and *HSP70*, known for their cytoprotective roles, were upregulated, reflecting their function in mitigating stress and preventing apoptosis. Genes for β-actin and β-tubulin were initially upregulated, promoting cytoskeletal adaptation to microgravity. However, their expression decreased with prolonged s-µ*g* exposure, indicating structural weakening. 

Chen et al. [76] explored the neural differentiation of MSCs under s-µ*g* (clinostat 4 h, 3 d, 7 d, 10 d) conditions. They found that cells cultured in a neural differentiation environment under s-µ*g* showed increased secretion of neurotrophins such as tyrosine hydroxylase (TH) and choline acetyltransferase (CHAT), as well as microtubule-associated protein 2 (MAP-2), nerve growth factor (NGF), brain-derived neurotrophic factor (BDNF), and ciliary neurotrophic factor (CNTF). These results demonstrated that s-µ*g* could stimulate the neural differentiation potential of MSCs. 

Clinorotation inhibited population growth of rBMSCs and their differentiation towards osteoblasts. Akt and extracellular signal-related kinase 1/2 phosphorylation levels and the expression of core-binding factor α1 decreased after 3 days of s-µ*g*. Dai et al. [77] demonstrated that clinorotation (24–96 h) disrupted key signaling pathways essential for MSC proliferation.

#### 4.1.3. Epidermal Stem Cells (EpSCs)

EpSCs are crucial for skin maintenance and repair and serve as a protective barrier against external damage. Recent studies have explored how µ*g* might influence the proliferation, differentiation, and metabolism of EpSCs, providing insights into potential applications for skin regeneration in space and on Earth. Lei et al. [78] conducted a study using an RCCS bioreactor and cocultured microcarriers with human EpSCs isolated from children’s foreskins. Over a 15-day period, they found that Ki67-positive cells, indicative of proliferation, accounted for 10% of EpSCs under s-µ*g*, significantly higher than under static 1*g* conditions. These data suggest that s-µ*g* facilitates EpSC proliferation. Cells in the RCCS revealed a low expression of involucrin at day 10. In addition, RCCS cells formed 3D multilayer epidermal structures [78].

Moreover, another study by Li et al. [79] investigated the effects of s-µ*g* (RCCS) on the metabolism of EpSCs, focusing on lipid metabolites. Lipids such as phosphatidylcholine (PC), phosphatidylethanolamine (PE), and phosphatidylserine (PS) are essential for cell membrane structure and various cellular functions, including proliferation, apoptosis, and energy metabolism. Using liquid chromatography–mass spectrometry (LC–MS), the study published an upregulation of PEs and a significant downregulation of PSs and PCs in hEpSCs cultured under s-µ*g* compared to 1*g* conditions. These results suggested that s-µ*g* weakened EpSC proliferation and increased apoptosis. Additionally, sphingolipids (SPLs), including ceramides (Cer) and sphingomyelin (SM), are vital for cell cycle regulation, senescence, apoptosis, and migration. The study noted a significant upregulation of SM levels and a reduction in Cer and its downstream metabolites under s-µ*g*, indicating an altered proliferation and differentiation.

#### 4.1.4. Cancer Stem Cells (CSCs)

CSCs represent a subpopulation within tumors with characteristics like adult stem cells, such as self-renewal, differentiation, and asymmetric cell division, and can initiate new tumors when implanted in animal models. Pisanu et al. [80] investigated lung CSCs derived from non-small cell lung cancer cells (H460) by analyzing CSC markers CD44, CD133, and ALDH. The study found lung CSCs lost stemness features under RPM exposure and showed increased apoptosis compared to controls. The expression of lung cancer protein markers ALDH, Nanog, and Oct-4 was downregulated. Similarly, Kelly et al. [81] used a NASA-developed hydrodynamic focusing bioreactor (HFB) to culture CSCs from various cancer cell lines. They found an increase in CD133+ CSCs under s-µ*g* compared to normal gravity, indicating that altered gravity stimulates CSC proliferation.

#### 4.1.5. Hematopoietic Stem Cells (HSCs)

Astronauts often experience symptoms such as anemia and hematopoietic disorders in space, prompting studies on the effects of µ*g* on HSCs. HSCs are characterized by CD34, crucial for hematopoietic activities in the bone marrow, with the potential to differentiate into various blood cells. Plett et al. [82] cultivated CD34^+^ cells in an RWV for 3 days and found a significant inhibition of migration potential mediated by stromal cell-derived factor 1 (SDF-1a). This inhibition was linked to a cytoskeletal disorder, evidenced by decreased F-actin formation and microfilament reorganization. Moreover, CD34^+^ cells in s-µ*g* exhibited lower proliferation rates than in normal gravity conditions. HSCs in s-µ*g* exit the G0/G1 phase more slowly and exhibit cell cycle arrest in this phase [83]. s-µ*g* also prolongs the S phase and reduces cyclin-A expression, which is essential for cell proliferation [82]. Recent studies suggest that the inhibition of cell proliferation in µ*g* is related to decreased activity of the stem cell factor (SCF) pathway. Analyses during spaceflight revealed a significant downregulation of the SCF receptor and increased expression of negative regulators of the SCF pathway, such as Dusp1, Dusp2, Ptpn1, and Ptpn2 [84]. This downregulation leads to cell cycle arrest by inhibiting the SCF pathway. r-µ*g* also affects HSC metabolism, suppressing pathways like ether lipid metabolism, unsaturated fatty acid biosynthesis, and glycolipid metabolism, resulting in dysfunctions that impact HSC proliferation [84]. Conversely, Liu et al. [85] observed that long-term RWV culture of HSCs with supplemented cytokines resulted in a 30-fold increase in CD34^+^ cells, suggesting some potential for HSC expansion under prolonged µ*g* conditions. Blaber et al. [86] demonstrated that r-µ*g* (15 days of spaceflight) induced significant bone resorption in rats, enlarging the bone marrow cavity and downregulating gene expression markers associated with hematopoietic differentiation. Histological analyses revealed decreased megakaryocytes and increased red blood cells, suggesting mechanisms behind spaceflight-induced anemia. Cao et al. [87] found that s-µ*g* in hindlimb unloaded (HU) mice reduced populations of natural killer (NK) cells, B cells, and erythrocyte precursors while increasing T cell and neutrophil populations. This study also noted changes in the expression of key regulatory molecules involved in HSC differentiation. Similarly, Dai et al. [88] reported increased proportions of macrophages, granular leukocytes, and monocytes, reducing mature erythrocytes and B lymphocytes, indicating altered differentiation pathways and potential immune dysregulation in space. Further studies showed HSCs’ ability to differentiate into vascular endothelial cells under µ*g*. Chiu et al. [89] cultured CD34^+^ cells in an RWV bioreactor with vascular endothelial growth factor, resulting in the formation of vascular tubular structures and endothelial phenotypic markers, highlighting the potential for stem cell therapy in vascular diseases.

Table 1 summarizes the most important data reviewed in this chapter. 

### 4.2. Peripheral and Central Nervous System 

It is well known that extended reductions in physical activity induced by prolonged bed rest or µ*g* can induce muscle deconditioning and that such changes impact other physiological systems [90,91]. One instance is the nervous system, which is negatively impacted by reduced physical activity, leading to a higher occurrence of neurological issues such as chronic pain. To gain mechanistic insight into transcriptional alterations that occur following muscle deconditioning, McFarland and coworkers conducted RNA sequencing (RNA-seq) analysis on muscle biopsies obtained from human donors participating in a 5-week bed rest study with an exercise intervention arm [92]. Bulk RNA-seq was performed on muscle samples from a previous bed rest investigation [93]. In total, 25 healthy, nonsmoking adult subjects aged 20–54 years were randomly assigned to either the bed rest-only group (1 female, 8 men) or bed rest with exercise countermeasure (2 female, 14 men).

The research unveiled 1352 differentially expressed genes (DEGs) in bed rest subjects without exercise intervention, compared to only 132 DEGs in subjects with intervention. By employing a computational approach to investigate the interactions between muscle and human dorsal root ganglion (DRG) neurons, the authors identified 591 upregulated muscle genes in the non-intervention group, of which 26 were ligands with receptors expressed by human DRG neurons. Notably, DRG neurons are pivotal in nociception and serve as generators of signals associated with chronic pain. The study identified a distinct splice variant of one such ligand, placenta growth factor (PGF), within the deconditioned muscle, which binds to neuropilin-1. This receptor is highly expressed in DRG neurons and is recognized for promoting neuropathic pain.

In conclusion, the study demonstrates that exercise intervention safeguards muscle against deconditioning-related transcriptomic alterations and mitigates changes in the expression of ligands that could sensitize DRG neurons or impact other cell types across the body. This study, which also marks one of the longest periods of human physical inactivity examined through RNA sequencing technology, incorporates this newfound understanding within the context of potential effects on DRG neurons [92].

The metabolomics profile of the secretome of space-flown human oligodendrocytes was investigated by the Espinosa-Jeffrey group [94]. Neurological impairments have been observed after human space journeys, including intracranial hypertension (ICP) and visual impairment intracranial pressure (VIIP). Hence, this interesting study aimed to investigate the impact of space and thus r-µ*g* on the metabolomics profile of oligodendrocyte progenitors (OLPs), constituting the myelinating cells of the central nervous system (CNS). The study revealed elevated levels of glutamate and enhanced energy metabolism during the 26-day space mission of OLPs. After spaceflight, OLPs exhibited markedly increased mitochondrial respiration and glycolysis. Furthermore, employing a global metabolomics approach, the Espinosa-Jeffrey group identified endogenous metabolites (related to important cellular pathways, including glycolysis, TCA cycle, glutamate metabolism, and lipid metabolism) secreted by OLPs while in space, which were significantly influenced by μ*g*.

Collectively, these findings offer valuable insights into the energetic status of OLPs during space missions and post-space flight. The molecular and functional significance of the identified pathways may thus present promising targets for future therapeutic interventions aimed at supporting human long-term space exploration missions [94].

In a recent paper, the Espinosa-Jeffrey group continued their effort to determine how space µ*g* impacts the CNS, and specifically how space-flown neural stem cells (NSCs) readapt to the gravity on Earth [95]. The study observed that most of these cells survived the spaceflight and exhibited self-renewal capabilities. However, some cells displayed heightened stress responses and exhibited behavior reminiscent of autophagy. Proteomic analysis was used to determine if molecules present in the secretome of space-flown NSCs were responsible for inducing these responses. Hence, naive, non-starved NSCs were exposed to a medium containing the secretome of space-flown NSCs. Remarkably, the proteomic analysis of the secretome identified the secreted protein acidic and rich in cysteine (SPARC) as the protein with the highest content produced by the space-flown NSCs. SPARC has been shown to induce endoplasmic reticulum stress, leading to cell death.

Taken as a whole, these findings provide novel insights into the response of NSCs to µ*g* environment in space. Specifically, identifying SPARC as potential µ*g* sensors may open avenues for future research aimed at targeting the metabolism of certain secreted proteins. The results also represent an initial step in identifying gravity-sensing molecules as targets for modulation and the development of effective countermeasures to mitigate intracranial hypertension in astronauts. However, further studies are needed to establish a direct relationship between microgravity-induced stress and SPARC as a potential marker [95].

Table 2 overviews the most important literature published in this field.

### 4.3. Eye

The eye is one of the most delicate and vital tissues in the human body. Consequently, it is not unexpected that more than 60% of the active astronauts returning to Earth after extended stays aboard the ISS experience neuro-ophthalmic alterations, also known as spaceflight-associated neuro-ocular syndrome (SANS). Although several papers have investigated spaceflight-induced alterations, including SANS, only a few studies are based on human cells [96].

**Table 2:** Omics studies on the nervous system and the eye under microgravity conditions.

Cells/Animals/ Humans	Omics	µ*g*	Device/Platforms	Refs.
Human subjects	RNA sequencing on muscle biopsies 1352 DEGs in bed rest subjects without an exercise intervention. 132 DEGs in subjects with the intervention. Among 591 upregulated genes in the no-intervention arm, 26 were ligands that have receptors that are expressed by DRG neurons a specific splice variant of PGF, which binds to neuropilin-1	s-µ*g*	Bed rest, 5 weeks	[92]
Oligodendrocyte progenitors	Metabolomics profile of oligodendrocyte progenitors. Inflight: increased glutamate and energy metabolism; significantly modulated endogenous metabolites Post-flight: increased mitochondrial respiration and glycolysis	r-µ*g*	Spaceflight, 26 d	[94]
Neural stem cells	The behavior of space-flown NSCs and their readaptation to 1*g* on Earth. Four-fold increase in stress responses. A high amount of secreted protein is acidic and rich in cysteine (SPARC).	r-µ*g*	Spaceflight, ISS for 39 d	[95]
ARPE-19 cells	R-µ*g*: no change in cell viability or apoptosis. Vimentin cytoskeleton remodeling changes in the transcriptomic profile: 23,556 genes with 5500 DGEs on ISS (vs. 1*g*), consistent with cell dysfunction adaptations. Coenzyme Q10 increased cell resistance to damage.	r-µ*g*	ISS, 4 d	[15]
Jurkat cells (T lymphocyte cell line)	3D cell culture mitigates the impact of the RPM on the transcriptome and nuclear irregularities of T cells compared to 2D cell culture. Activated T cells are less affected by the RPM than resting T cells. RPM: effects on circulation T cells, including enrichment of genome loci, especially on chromosomes 1 and 19, reduced TCR expression, diminished T cell activation, increased DNA damage, and transcriptome alterations.	s-µ*g*	RPM, 24 h resting stage, and activating cells	[97]
Rodent models, Astronaut samples	Role of circulating microRNAs as potential biomarker for health risks. Confirmation of previously identified miRNAs that regulate rodent responses to spaceflights in miRNA seq, single-cell RNA-seq, and single-cell assay for transposase accessible chromatin. Significantly, a subset of the miRNAs discovered in the investigation (miR-125, miR-16, and let7a) was observed to govern vascular injury induced by simulated deep-space radiation. Countermeasures to mitigate the damage caused to the body by the space environment	r-µ*g*, s-µ*g*	HU, irradiation mice, and rats	[98]
Platelets from healthy men	Distinct platelet miRNA signature, elevated platelet count, and plateletcrit. Changes correlating with a distinctive circulating protein biomarker pattern. HSP27, LOX-1, and DKK1 were identified. Platelets are indicators and functional biomarkers of epigenetic changes within the cardiovascular system.	s-µ*g*	dry immersion (DI) model, 12 healthy men, 3 d	[99]
24 healthy participants	Measurement of creatine kinase activity, GDF-8/myostatin; slow skeletal muscle troponin T; prostaglandin E2), neurotrophic factors (BDNF; GDNF) and C-terminal agrin fragment (CAF); no significant effect of bed rest or sex on any of these parameters	s-µ*g* 1*g*	60 d, AGBRESA bed rest study with or without daily 30 min continuous/intermittent centrifugation with 1*g*	[100]

A prominent example is a recent paper by Cialdai et al., who investigated the cellular and molecular effects induced in human adult retinal pigment epithelium (ARPE-19) cells after three days of incubation aboard the ISS [15]. There were no observed changes in viability or apoptosis during the phase of µ*g*, and consistently with previous research, Cialdai et al. detected cytoskeletal remodeling after exposure to μ*g* conditions. Remarkably, the authors noted a significant alteration in the vimentin network, manifested by the migration of vimentin from the cell surface to perinuclear regions in ARPE-19 cells cultured for three days onboard the ISS. Redistribution of vimentin may suggest modifications in cellular morphology and the ability for inter-cellular interactions. Exposure to spaceflight conditions also revealed that ARPE-19 cells exhibited aggresome-like structures, suggesting a likely association between microgravity and altered protein processing in these cells.

The detected changes in cell morphology in the ARPE-19 cells cultured onboard the ISS were accompanied by notable modifications in the transcriptome profile [15]. Hence, among the 23,556 genes investigated, over 5500 exhibited differential expression in the environment of the ISS compared to ground controls. Furthermore, assessment of the quantity of active microRNAs (miRNAs) and dysregulated long non-coding RNAs (lncRNAs) showed that among 366 screened miRNAs, 19 displayed differential downregulation of target genes, and over 250 long lncRNAs were deregulated in ARPE-19 cells cultured onboard the ISS. Intriguingly, predictive analysis indicated that approximately 100 pathways were significantly influenced following spaceflight, with a focus on pathways related to cellular responses to adaptation or damage caused by the space environment. Using gene ontology (GO) analysis, the authors demonstrated that the incubation of ARPE-19 cells in reduced gravity affected several important cellular mechanisms, including ion binding and the ability to respond to the presence of unfolded proteins, which are indicative of adaptive cellular dysfunction [15].

Interestingly, the study also aimed to assess the effect of coenzyme Q10—an antioxidant with antiapoptotic properties—on gene expression in ARPE-19 cells. They identified 153 differentially expressed genes in coenzyme Q10-treated cells incubated onboard the ISS compared to untreated controls. Twenty-two pathways were significantly affected, including TGF-beta signaling, Hippo signaling, p53 signaling, the senescence pathway, deregulation of protein processing in the endoplasmic reticulum, and mitophagy clearly denoting coenzyme Q10 as a putative countermeasure against SANS, but further examination is needed to fully unravel the potential of this strategy [15].

### 4.4. Cardiac Cells 

The cardiovascular system is known to be impacted by spaceflight conditions such as µ*g* and space radiation. Astronauts in space experience a redistribution of blood and other body fluids in the direction of the upper body that triggers a series of adaptation mechanisms in the cardiovascular system [101]. Such changes include cardiac output and stroke volume and decreased ventricular size, mean arterial pressure, and systemic vascular resistance. These adaptation mechanisms often lead to cardiovascular deconditioning and post-flight orthostatic intolerance (OI) [7,102]. While these systemic changes have been thoroughly characterized, the cellular and molecular effects underlying these changes remain understudied and may be critical to understanding and treating spaceflight-derived cardiovascular conditions [103]. Moreover, although a few omics studies of cardiac cell populations under spaceflight or s-μ*g* conditions have been reported, this field remains understudied. Exposure of human stem cell-derived cardiomyocytes (CMs) to altered gravity conditions in combination with omics technologies uncovered a µ*g*-controlled axis causing contractile dysfunctions to CMs via the induction of senescence processes [104].

#### 4.4.1. Human Aortic Smooth Muscle Cells

Among the group of cardiac cells for whom omics studies have been reported are human aortic smooth muscle cells (HASMCs). Scotti et al. performed an RNA-seq analysis of HASMCs cultured on spherical microcarrier beads for three days aboard the ISS [103]. GO analysis revealed that most DEGs were related to extracellular processes and the extracellular matrix (ECM). Also, the Gene Annotation Database (GAD) analysis revealed associations between the DEGs and metabolic, cardiovascular, cancer, immune, and hematological diseases.

Markers of the three main phenotypic profiles of the HASMCs (contractile, synthetic, and osteogenic) were downregulated, including smooth muscle alpha-actin (αSMA), matrix metalloproteinases (MMPs), and bone morphogenic proteins (BMPs). This is relevant given that under normal 1*g* conditions, vascular smooth muscle cells retain their contractile phenotype and only make a phenotypic switch under vascular damage or disease [105,106]. However, the downregulation of the contractile apparatus was not followed by an increased expression of markers corresponding to synthetic or osteogenic phenotypes. This result is also consistent with the observed decrease in vascular tone and cardiovascular deconditioning in astronauts aboard the ISS [7].

A transcriptomic analysis searching for potential pathway regulation mechanisms showed that thirty canonical pathways, including STAT3, NFκB, PI3K/AKT, HIF1α, and endothelin pathways, were significantly affected. Three signaling pathways related to cardiac hypertrophy were also shown to be significantly affected [103]. The STAT3 pathway is reported to play a role in the phenotype-switching process of VSMCs and is inversely related to the production of the contractile apparatus components [107]. Surprisingly, several of its genes were downregulated, suggesting that other molecules or pathways may be involved in the contractile apparatus downregulation. Several genes of the NFκB pathway were also downregulated, suggesting the activation of an anti-inflammatory process during spaceflight conditions. Other changes in gene expression were related to reductions in proliferation, survival, wound healing, migration, and angiogenesis.

#### 4.4.2. Cardiovascular Progenitor Cells

Various studies have shown that spaceflight conditions enhance stemness or the differentiation of stem cells towards specific phenotypes [108,109,110]. Cardiovascular Isl-1+ progenitor cells (CPCs), resident to the heart, have been studied under s- or r-µ*g* conditions. The results show that CPCs respond in an age-dependent manner to the µ*g* stimuli and result in different differentiation outcomes. For example, Fuentes et al. exposed neonatal and adult CPCs to six or seven days of s-μ*g* via 2D clinorotation [111]. The results demonstrated an increased expression of DNA repair genes (RAD50, RAD23) and stemness-associated genes (MESP1, Oct-4, Brachyury) in neonatal CPCs that was not observed in adult CPCs. MicroRNA (miRNA) profiling of neonatal CPCs under s-µ*g* also showed that miRNAs known to play a role in differentiation (microRNA-99a-5p and microRNA-100-5p) were downregulated. Finally, neonatal CPCs under s-μ*g* conditions showed decreased tube formation and von Willebrand factor (vWF) expression, markers of endothelial differentiation. Overall, these data indicate an enhanced stemness state in neonatal CPCs after exposure to s-μ*g*, which has been observed in other cell types [108,112]. In contrast to this, adult CPCs under s-μ*g* showed an increase in both tube formation and vWF expression in addition to an increased expression of troponin T (Trop T) and myosin light chain 2 (MLC2v), which are signs of differentiation along the cardiomyocyte linage [113].

These results were confirmed under real spaceflight conditions. Neonatal and adult CPCs were cultured for 12 and 30 days aboard the ISS and compared to 1*g* controls [114,115]. Gene expression analysis, miRNA profiling, and a tube formation assay showed an enhanced stemness state in neonatal CPCs after 12 days of culture aboard the ISS. Among the genes that were upregulated in neonatal CPCs were genes known to play a role in embryonic stem cell self-renewal (*POU5F1*, *NANOG*, *SOX2*), DNA repair genes (*RAD23*, *RAD50*), mesodermal specification (FOXC1, MESP1), and early cardiogenesis (*GATA4*, *KIT*, *PDGFRA*, *ISL1*, *KDR*) [115]. Early precardiac mesoderm development transcripts *BMP4*, *TBX3*, *TBX5*, and *TBX18* were also increased in neonatal CPCs under spaceflight conditions. microRNA-99a-5p and microRNA-100-5p downregulation and reduced tube formation capability of neonatal CPCs aboard the ISS were observed under s-μ*g* also. 

Surprisingly, transcriptomic analyses and miRNA profiling of adult and neonatal CPCs after 30 days aboard the ISS showed an enhanced stemness state for both cell lines [114]. Moreover, cell cycle progression, differentiation, cardiogenesis, oxidative stress protection, and focal adhesion transcripts were upregulated independently of age. These results aligned with miRNA expression analysis, which showed an upregulation of miRNA-17–92 cluster elements and increased proliferation. The miRNA-17-92 cluster has been reported to promote cell cycle progression, proliferation, and oxidative stress protection [116,117].

Human-induced pluripotent stem cell-derived cardiac progenitors (HiPSC-CPCs) have also been studied aboard the ISS. Hwang et al. cultured 3D HiPSC-CPCs for three weeks aboard the ISS and performed RNA-sequencing analysis [29]. Results showed that under spaceflight conditions, there is an increased expression of genes related to cardiac function (*THBD*, *GADL1*), contraction *(MYL2*, *TNNI3*, *SCN5A*, *RYR2*), conduction (*ATP2B4*, *PRKACA*, *ANK2*), proliferation, and cardiac differentiation (*CCBE1*, *CCND2*, *IGFBP5*, *BDKRB2*). On the other hand, there is a decreased expression of genes related to ECM regulation (*ITIH5*, *COL4A4*, *PTPR21*, *COL26A1*, *HPX*) and focal adhesion (*ITGA11*, *PROCR*, *ANXA1*, *LIMA1*). These results suggest that spaceflight conditions may benefit the growth and differentiation of 3D HiPSC-CPCs. Cardiac progenitor cells show different responses to spaceflight conditions depending on age and source. Further understanding of the pathways and mechanisms involved in the response of CPCs to spaceflight conditions may be beneficial for biomedical space research and novel stem cell development protocols.

#### 4.4.3. HiPSC-Derived Cardiomyocytes

HiPSC-derived cardiomyocytes (HiPSC-CMs) have recently emerged as a possible solution to the limited availability and long-term maintenance of cardiomyocytes for heart therapy and disease modeling [118]. For this reason, many studies have focused on using this cell type to analyze the effects of µ*g* on human cardiac physiology. Wnorowski et al. cultured HiPSC-CMs for 5.5 weeks onboard the ISS and compared them to ground and postflight samples regarding gene expression, structure, and function [119]. RNA-sequencing analysis revealed 2635 DEGs among flight, post-flight, and ground control samples. Upregulation in genes involved in the mitochondrial metabolism pathway (KLF5, COUP-TFII) showed the most changes between flight samples and ground control [120,121]. These results coincide with previous studies showing heart mitochondrial adaptation after exposure to microgravity conditions [122,123]. Transcriptomic and proteomic analyses of HiPSC-CMs exposed to 2D clinorotation have also shown that 48 h is enough time to induce mitochondrial structural and functional changes, causing contractile dysfunctions to CMS via the induction of senescence processes [104].

Although flight samples retained their sarcomeric structure and morphology compared to 1*g* controls, exposure to spaceflight conditions influenced Ca^2+^ handling, leading to a decreased Ca^2+^ recycling rate and beating irregularity. These results regarding sarcomeric structure and Ca^2+^ handling were also in accordance with results on HiPSC-CMs exposed to s-μ*g* via 2D clinorotation [104]. Gene expression analysis revealed no significant change in the expression levels of Ca^2+^ cycling-related genes, suggesting that these changes were due to stress-induced Ca^2+^ sarcoplasmic reticulum load increase and possibly RyR2 leakage. Additionally, hypertrophy-associated genes such as myocyte enhancer factor 2D (MEF2D), cardiac troponin T, and troponin 11 were significantly upregulated in the flight samples but not in the post-flight samples compared to the ground controls. Moreover, while two-group comparisons revealed 3008 DEGs between ground and flight samples, there were only 1049 DEGs between post-flight and ground samples. These results suggest that the hiPSC-CMs adopt a reversible gene expression profile under spaceflight conditions.

A reduction in Ca^2+^ recycling was likewise observed by Han et al. in HiPSC-CMs cultivated aboard the CSS for a period of six days [124]. However, they also detected changes in sarcomeric structure, such as reduced sarcomeric length and cTNT content not observed in previous studies. An effect accompanied this on thiamine utilization. The authors performed a metabolomic analysis of the cell culture supernatant. They found changes in metabolites related to the sulfur relay system, thiamine metabolism, vitamin digestion and absorption, and ABC transporters [124]. From this, it was suggested that µ*g* affects normal thiamine uptake and utilization. Furthermore, thiamine supplementation reversed some effects observed under µ*g* conditions, such as beating rate, sarcomeric length, cTNT content, and Ca^2+^ handling. The transcriptional analysis also revealed DEGs related to lipid metabolism, metabolism of cofactors and vitamins, and carbohydrate metabolism.

Table 3 summarizes the current knowledge about cardiac cells grown under µ*g* conditions.

### 4.5. Vascular System

Exposure to the spaceflight environment results in substantial alterations to the cardiovascular system. For endothelial cells (ECs), it has been shown that µ*g* impairs nitric oxide synthesis, cell adhesion, extracellular matrix composition, cytoskeleton organization, cytokines, and growth factor secretion. 

Using a 3D clinostat, human endothelial cells (EA.hy926 cell line) were investigated for up to 10 days in s-µ*g*. The maximal gene upregulation occurred after 10 min of clinorotation. Also, caspase-3, bax, and bcl-2 proteins were increased after 10 days of s-µ*g*, and a reduction in release of brain-derived neurotrophic factor, tissue factor, vascular endothelial growth factor (VEGF), and endothelin-1 was observed [126]. By using clinorotation for 24 h, an increase in the inducible nitric oxide synthase (iNOS) expression was observed in human umbilical vein endothelial cells (HUVECs) by a mechanism dependent on suppression of AP-1 [127]. s-µ*g* created by a clinostat (MG-3) for 72 h induced apoptosis of human pulmonary microvascular endothelial cells (HPMECs), and the effect was shown to be correlated with a downregulation of the PI3K/AKT pathway, increased expression of NF-kB, and depolymerization of F-Actin [128]. Another study used a clinostat to investigate the interactome of miRNAs and transcriptome of HUVEC cells exposed to short-term s-µ*g*. The miRNAs hsa-mir-496, hsa-mir-151a, hsa-miR-296-3p, hsa-mir-148a, hsa-miR-365b-5p, hsa-miR-3687, hsa-mir-454, hsa-miR-155-5p, and hsa-miR-145-5p differentially regulated the genes involved in cell adhesion, angiogenesis, cell cycle, JAK-STAT signaling, MAPK signaling, nitric oxide signaling, VEGF signaling, and wound healing pathways [129].

**Figure 3:**
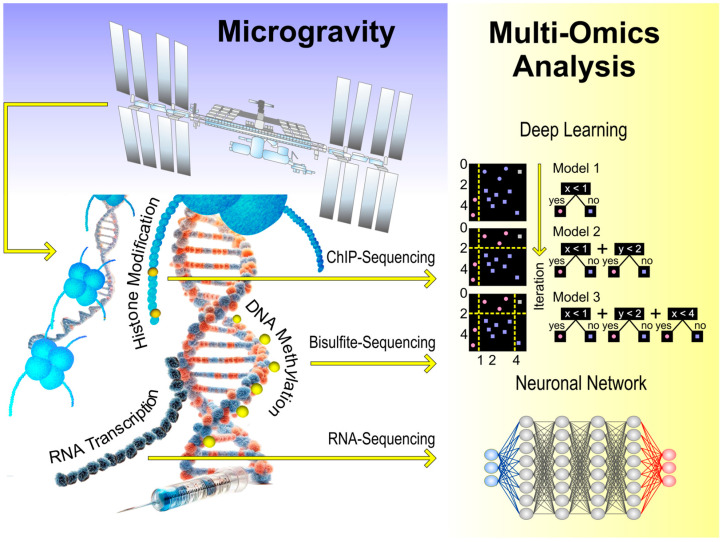
Concept of an integrative multi-omics AI analysis of transcriptional and epigenetic consequences of microgravity exposure.

Conditions of s-µ*g* (RPM) induced changes in the secretome of HUVEC cells. S-µ*g* altered the secretion rate of proteins involved in the regulation of the cytoskeleton assembly and decreased the secretion of key pro-inflammatory cytokines (IL-1α and IL-8), and the pro-angiogenic factor bFGF. S-µ*g* also increased the secretion of chemokines (Rantes and Eotaxin), which participate in leukocyte recruitment [130]. A gene expression array analysis was conducted on EA.hy926 cells following 5 and 7 days of RPM exposure (Figure 3). RPM treatment led to the development of 3D cell aggregates and resulted in a minimum four-fold alteration in the expression of 27 genes compared to cells maintained under 1*g* conditions. IL-8 (interleukin-8) and VWF (von Willebrand factor) were among the most prominently affected [18]. EA.hy926 cells and human microvascular endothelial cells (HMVECs) were cultured in the RPM environment for 5 and 7 days. HMVECs exhibited accelerated 3D structure formation compared to EA.hy926 cells. Proteomic analysis of these rapidly developing, tube-like HMVEC structures revealed a transient increase in ribosomal protein levels during assembly [131]. Transcriptomic analysis of HUVEC cells exposed to s-µ*g* within the RPM for 24 h identified differential expression of 177 genes. Among the upregulated genes, distinct clusters associated with cellular responses to external stimuli and cell motility and proliferation were evident. In the downregulated genes, clusters of transcription factors with “zinc finger” domains and factors involved in regulating morphogenesis and angiogenesis were distinguished [132]. Additionally, a 35-day long-term RPM experiment demonstrated angiogenesis in EA.hy926 endothelial cells. The factors VEGF, NGAL, IL-6, IL-8, MCP-1, VCAM-1, ICAM-1, fibronectin, and RANTES were shown to be affected during long-term s-µ*g* exposure [133].

The study revealed a molecular architecture linking energy metabolism and immunodeficiency in microgravity [134]. EA.hy926 cells were exposed to the International Space Station (ISS) environment for 12 days. r-µ*g* conditions induced the formation of 3D cell aggregates and upregulated collagen and laminin protein expression. The supernatant analysis unveiled alterations in the secretion of several growth factors, cytokines, and ECM components compared to cells cultivated at 1*g* or on the RPM [135]. Another study investigated the combined effects of space radiation with µ*g* and included a 1*g* control in the spaceflight environment. DNA repair mechanisms were found to be activated in µ*g* and the transcriptomics analysis showed the opposite effects of µ*g* and space radiation: the radiation exposure stimulated pathways for hypoxia and inflammation, DNA repair and apoptosis, and inhibition of autophagic flux (aged-like phenotype). µ*g*, on the contrary, activated pathways for metabolism and proliferation [136]. Finally, a study involves RNA-seq analyses on human aortic smooth muscle cells (HASMCs) cultured for 3 days in r-µ*g* (ISS). Transcriptomic analysis revealed alterations in 4422 genes, including the downregulation of contractile, synthetic, and osteogenic markers such as smooth muscle alpha-actin (αSMA), matrix metalloproteinases (MMPs), and bone morphogenic proteins (BMPs). Also, the signaling pathways STAT3, NFκB, PI3K/AKT, HIF1α, and endothelin were affected [103].

A gene array analysis was conducted on EA.hy926 endothelial cells under real microgravity (r-µ*g*) conditions during a parabolic flight to investigate the effects of short-term microgravity exposure. The gene array analysis revealed 320 significantly dysregulated genes after the first parabola (P1) and P31. Several gravisensitive signaling elements, such as AMPKα1 and integrins, are involved in the reaction of ECs to altered gravity [137]. HUVEC cells were exposed to a 10-day spaceflight to examine samples exposed to space conditions. A microarray analysis revealed 1023 significantly modulated genes: the thioredoxin-interacting protein was the most upregulated (33-fold), and heat-shock proteins 70 and 90 were the most downregulated (5.6-fold). Ion channels (*TPCN1*, *KCNG2*, *KCNJ14*, *KCNG1*, *KCNT1*, *TRPM1*, *CLCN4*, *CLCA2*), mitochondrial oxidative phosphorylation, and focal adhesion were also affected, and the authors conclude that µ*g* affects the same molecular machinery for sensing flow alterations [138]. To investigate the impact of microgravity on immune function, human endothelial cells were challenged with lipopolysaccharide (LPS) during spaceflight. The transcriptomic analysis revealed suppression of *Lbp*, *MyD88*, and *MD-2*, which encode proteins responsible for early LPS uptake. IL-6 and IL-8 surged in response to LPS insult in µ*g*. Finally, contrasting proteomic expressions of B2M, TIMP-1, and VEGRs suggested impaired pro-survival adaptation and healing mechanisms. Differential expression of miR-200a and miR-146b suggested the susceptibility of hosts in spaceflight to oxidative stress [139]. The metabolite profile of space-flown endothelial cells was analyzed to complement this study. The cells in space and on the ground were exposed to lipopolysaccharide (LPS), and then acids, proteins, and metabolites were collected 4 and 8 h post-LPS exposure. Several markers linked to energy deficiency, amino acids such as tryptophan, creatinine, dopamine, and glycine, and cofactors such as lactate and pyruvate were altered. Table 4 summarizes the most important papers available in this research field.

### 4.6. Circulation

Focusing on the impact of s-µ*g* on the immune system, especially in circulating and tissue-resident T cells, ElGindi and coworkers employed both 2D cell culture and 3D collagen matrices [97]. T cells, which are pivotal components of the adaptive immune system and long-term immunity, circulate in the blood and reside within tissues. To date, studies exploring the effects of µ*g* on T cells have primarily focused on peripheral blood or traditional 2D cell culture systems, which mimic blood circulation. However, 3D cell culture techniques were employed to better replicate tissue environments, providing physiologically and pathologically relevant models.

T cells were examined in resting and activated states (using phorbol 12-myristate 13-acetate and ionomycin) following incubation at µ*g* conditions, using an RPM, or at 1*g*. The study indicates that 3D cell culture mitigates the impact of s-µ*g* on T cells’ transcriptome and nuclear irregularities compared to 2D cell culture. Intriguingly, activated T cells appear less affected by simulated microgravity than resting T cells. Collectively, the findings suggest that µ*g* conditions result in several effects on the circulation of T cells, including enrichment of genome loci, especially on chromosomes 1 and 19, reduced T cell receptor (TCR) expression, diminished T cell activation, increased DNA damage, and altered the transcriptome. Interestingly, fewer effects of microgravity on resting and activated T cells were observed in tissue-resident T cells. Further studies are needed to ascertain the cause of such differences between circulating and tissue-resident T cells. Importantly, the provided data offer a new understanding of the impact of simulated microgravity on both circulating and tissue-resident T cells, with potential implications for the health of future space explorers [97]. 

In 2020, Malkani and coworkers published a comprehensive paper investigating the spaceflight signature of circulating microRNA (miRNA) [98]. The study identified and validated a spaceflight-associated miRNA signature shared by rodents and humans in response to simulated microgravity and short-duration and long-duration spaceflight. Using astronaut samples from the NASA Twins Study [123], Malkani and coworkers confirmed previously identified miRNAs that regulate rodent responses to spaceflights in miRNA sequencing, single-cell RNA-seq, and single-cell assay for transposase-accessible chromatin. Significantly, a subset of the miRNAs discovered in the investigation (miR-125, miR-16, and let7a) was observed to govern vascular injury induced by simulated deep-space radiation. Antigomirs inhibiting the key spaceflight-associated miRNAs successfully rescued simulated deep-space-radiation-mediated damage in human 3D vasculature constructs, thereby demonstrating the physiological relevance of the identified circulating miRNA spaceflight profile. In this way, the study reveals targets for countermeasure development [98].

Twomey and coworkers investigated the µ*g*-induced miRNA signature in platelets obtained from 12 healthy non-athletic men aged 26–39 [99]. In this setting, the authors employed µ*g* conditions simulated by dry immersion as a state-of-the-art approach to deepen our understanding of the causes of chronic age-related conditions like cardiovascular disease. The effect of µ*g* conditions on the platelet phenotype was investigated in the subjects exposed to 3-day dry immersion (DI) to replicate most physiological impacts on the human body associated with r-µ*g*. 

A significant increase in platelet count, platelet crit, platelet adhesion, aggregation, and a modest platelet reactivity index (PRI) elevation was observed. The research identified 15 protein biomarkers and 22 miRNAs whose expression levels changed following DI [99]. Furthermore, a 3-day DI led to developing a prothrombotic platelet phenotype characterized by a distinct platelet miRNA signature, elevated platelet count, and platelet crit. These changes correlated with a distinctive circulating protein biomarker pattern. Hence, important proteins influenced by DI, including heat shock protein 27 (HSP27), lectin-like oxidized LDL receptor (LOX-1), and Dickkopf-related protein (DKK1), were identified. Collectively, these results emphasize platelets’ role as sensitive adaptive indicators and functional biomarkers of epigenetic changes within the cardiovascular system [99].

Artificial gravity has been used as a potential countermeasure to mitigate the effects of weightlessness during long-term spaceflights. The 60-day Artificial Gravity Bed Rest—European Space Agency (AGBRESA) study, a joint project of ESA and NASA, investigated the impact of bed rest with or without daily 30 min continuous (cAG) or intermittent (iAG) centrifugation with 1*g* at the center of mass [100]. In total, 24 healthy participants (8 women and 16 men) were enrolled in two consecutive campaigns in 2019 with 12 individuals each. The participants were divided into 3 groups (8 control groups, 8 cAG, and 8 iAG), and fasting blood samples were obtained before and on days 6, 20, 40, and 57 during 6° head-down tilt bed rest. Creatine kinase activity was determined by colorimetric enzyme assay, and ELISA-based assays were used to access the concentrations of circulating markers of neurotrophic factors (brain-derived neurotropic factor [BDNF], glia cell line-derived neurotrophic factor [GDNF]), C-terminal agrin fragment [CAF], and muscle wasting growth differentiation factor 8 [GDF-8]/myostatin, slow skeletal muscle troponin T, and prostaglandin E2).

Artificial gravity did not lead to alterations in neurotrophic factors like GDNF, CAF, or BDNF, nor in circulating markers of muscle wasting. This suggests it is a safe intervention without adverse effects on neuromuscular integrity. The absence of a significant effect based on sex or interactions based on sex suggests that this was not a prominent concern. The authors, therefore, advocated that a 60-day period of head-down tilt bed rest, with or without artificial gravity, has no impact on the neuromuscular secretome [100]. However, since the study presented high variation in a relatively small sample size, further investigations are needed to substantiate the findings.

### 4.7. Hematological System

The human hematological system, or the blood system, is a vital and intricate network within the body responsible for carrying out essential functions. It includes blood, bone marrow, and lymphatic organs such as the spleen, thymus, and lymph nodes. This complex network is crucial in transporting oxygen, nutrients, and waste materials, healing wounds, defending against infections, and maintaining internal balance [140,141,142,143]. The lack of gravity during spaceflight can significantly impact the body’s blood and immune systems [144,145]. Immune cells like T cells, B cells, and natural killer (NK) cells may not function normally, and there can be changes in cytokine secretion and cell signaling pathways [146,147]. Blood cell production and function, including red blood cells, platelets, and white blood cells, may also be affected [146,147,148]. Prolonged exposure to µ*g* can alter bone marrow and lymphatic system function. The impact of µ*g* may differ based on factors such as the duration of space travel and individual characteristics. Scientists are researching ways to counteract these effects, such as exercise, nutrition, and potential medications, to support astronaut health during long space missions [149].

This chapter offers a detailed examination of the most significant research undertaken within the last thirty years to elucidate the impact of µ*g* on various components of the human immune system.

Across various space missions, there is a consistent pattern of immune system effects associated with the µ*g* environment, including immune exhaustion, aging of the immune system, and increased inflammation. Analyses of blood samples collected from male and female astronauts during shuttle missions have revealed higher counts of white blood cells and granulocytes, impaired function of virus-specific T cells during and after spaceflight, and elevated levels of inflammatory markers such as TNF-α, IFN-α, and IFN-γ in blood plasma collected during spaceflight, indicating ongoing inflammation [150]. In a short-duration mission, analysis of gene expression in blood samples after the mission compared to baseline levels showed changes in pathways related to oxidative stress and cellular repair. The decrease in the antioxidant gene GPX1 suggests that higher levels of reactive oxygen species could induce immune responses in the space environment [151]. At the end of space missions, observations have shown increased oxidative stress in neutrophils and monocytes, suggesting disrupted mechanisms for managing oxidative stress in the space environment [152,153]. Astronauts on more extended missions, lasting 4 to 6 months, have displayed elevated mitochondrial dysfunction, which contributes to increased oxidative damage and inflammation, mirroring characteristics of immune aging [154].

Furthermore, astronauts on missions lasting up to 1 year have shown signs of accelerated aging, as evidenced by oxidative stress-induced DNA damage in peripheral blood mononuclear cells (PBMCs), shorter telomeres, lower telomerase activity, and increased chromosomal abnormalities compared to ground controls, persisting for up to 6 months after returning from spaceflight [155,156]. A study using two sounding rockets and a Soyuz spacecraft examined the effect of spaceflight on immune cell signaling triggered by lipopolysaccharide (LPS) stimulation in peripheral blood monocytes. These results were compared to tests conducted on 1*g* controls loaded onto an onboard centrifuge. The findings revealed that µ*g* inhibits LPS-dependent Jun-N-terminal kinase activation in contrast to the unaffected LPS-dependent activation of p38 Mitogen-Activated Protein Kinase [157].

Limited research has been conducted on the impact of µ*g* on dendritic cells (DCs) and their development. DCs are crucial in presenting antigens to T-lymphocytes and are essential for initiating an effective immune response. One study aimed to investigate whether the development of DCs from the monocytes of astronauts changed after spaceflight. Blood samples were collected from a crew member of the Eneide mission shortly after the flight, a longer time after their return to Earth, and one year after the mission. Monocytes were used as precursors to generate DCs. The cells were cultured alone in granulocyte-macrophage colony-stimulating factor (GM-CSF) and interleukin-4 (IL-4) for six days. They were then further stimulated for 24 h with a cocktail of cytokines. The differentiation was assessed by flow cytometry and immunofluorescence, examining the expression of typical DC markers. Gene expression was analyzed using RT-PCR, which showed downregulation of the genes encoding the following markers: *CD40*, *CD80*, *CD83*, *CD86*, and *NF-κB* in post-mission samples compared to the control sample, which showed high expression of the same genes [158].

The impact of extended periods in space on human health is intricate and necessitates a comprehensive understanding. In a groundbreaking study, researchers examined the health impact of a year-long mission aboard the ISS using genetically identical twin astronauts. One twin was in space, while the other was a matched ground control. The study involved a multifaceted analysis, providing insights into various aspects of immune function and epigenetic regulation during spaceflight. Gene expression analysis revealed significant changes in immune-related pathways across all cell types during spaceflight, affecting the adaptive immune system, innate immune response, and natural killer cell-mediated immunity [123].

Moreover, a DNA methylation analysis focused on the promoters of genes associated with T-cell differentiation and activation in CD4 and CD8 cells, uncovering differences between samples taken before, during, and after the mission. Notably, the analysis of cytokine data showed significant variations in cytokine levels before, during, and after the spaceflight, with specific cytokines exhibiting unique patterns. Interestingly, T cell receptor (TCR) repertoires remained consistent during spaceflight compared to before and after the mission, even after influenza immunization. These findings highlight the impact of long-duration spaceflight on immune-related pathways, epigenetic regulation of T cell function, inflammation signatures, and TCR repertoires. Understanding these changes is crucial for developing targeted strategies to reduce potential health risks during future human space exploration missions [123].

However, these studies are limited due to the infrequency of spaceflights, limited space on spacecraft, and high costs. Several studies have been conducted to check the impact of s-µ*g* on humans in vivo. These studies depended on head-down tilt bed rest (HDTBR) to simulate µ*g* and mimic how fluid shifts in the body during spaceflight [159]. For example, in a study, male participants exposed to HDTBR for five days experienced a decrease in certain types of immune cells and reduced leukocyte adhesion molecule (CD62L) expression. However, these levels returned to normal by the fifth day, and full recovery was observed by the fourth day after exposure. The study also found that specific signaling pathways related to immune response were activated, while others appeared impaired during the s-µ*g* exposure. Specific immune cells were activated early on, even without the typical signs of inflammation, possibly due to the mechanical stress caused by fluid redistribution [160]. Another study involving six male volunteers who underwent 120 days of HDTBR found changes in their stress hormone and immune cell profiles [161]. Interestingly, the expression of a particular insulin receptor on immune cells doubled by day 65 of the study and returned to near-normal levels during the recovery period. A critical immune signaling molecule, IL-6, was also significantly elevated at day 65, indicating increased immune activity during the s-µ*g* exposure [161]. 

Another science team measured reduced levels of specific immune signaling molecules, suggesting an impaired immunity to pathogens or tumors at day 21 of s-µ*g* exposure [162]. However, it is worth noting that all studies discussed indicated a rapid re-adaptation and recovery of the immune system within one week, regardless of the duration of exposure to s-µ*g*. In addition to dysregulated inflammation, publicity to s-µ*g* has been proven to affect molecular-mediated immunity at some stage in and after publicity. Eight male participants (elderly 26 and 38) were subjected to 21 days of HDTBR. The consequences indicated reduced blood Th1 cytokines IL-2, IFN-γ, and TNF-α tiers at day 21, returning to baseline tiers at recovery (6 days post-HDTBR). Since Th1 cells and cytokines set off phagocytic features of macrophages and DCs and play a widespread function in directing molecular-mediated immunity, a deficiency in vital Th1 cells/cytokine manufacturing during HDTBR indicates impaired immunity to a pathogen or tumor challenge. These findings imply that T-anergy might also expand at some stage in HDTBR [162].

Several studies were conducted in vitro to study the effect of s-µ*g* on the immune system. However, studies on the innate human immune system are limited. Neutrophil activation through reactive oxygen species (ROS) and myeloperoxidase expression multiplied after RCCS, suggesting moderate inflammatory responses have been engaged in s-µ*g*. It is known that neutrophils form 70% of the human leukocytes. Antioxidant remedy partly mitigated the inflammatory reaction, suggesting antioxidant remedy can be a precious countermeasure for destiny research [163]. Circulating monocytes and tissue-precise macrophages were significantly studied inside the µ*g* environment, revealing molecular subtype and analog-precise effects. M1 (proinflammatory) polarized macrophages displayed multiplied IL-6, helping tremendous immune profiles. In contrast, M0 and M2 macrophages did not express this increase. Various macrophage subtypes show extended or reduced proinflammatory cytokine expression stages, depending on the experimental platform and the cell type [164,165,166].

Research has extensively explored adaptive immunological cells in µ*g* [145]. When exposed to 2D clinorotation (60 rpm) for 5 and 60 min, primary human CD41 T cells exhibited a reduced abundance of CD3 and ZAP70 after 5 min and reduced IL-2R after 60 min. This indicates compromised T-cell signaling, which diminishes cell proliferation in microgravity [167].

After subjecting primary human T cells to RCCS simulation at 14 rpm for up to 90 min, the signaling transduction pathways of diacylglycerol in CD41 T cells were assessed to examine the downstream T cell stimulation pathways of the TCR (CD3) signaling components. The results showed no impact on downstream pathways, suggesting that s-µ*g* inhibits T-cell activation by modulating the T-cell response rather than the TCR signal itself [168,169]. 

The modulation of inhibitory signaling and other intricate regulatory pathways may play a role in this phenomenon. Consequently, there was a reduction in cell proliferation following a 24 h µ*g* simulation using an RCCS at ten rotations per minute (rpm), as evidenced by decreased human lymphocyte activity. This led to decreased IgM and IgG antibody production at 72 h, which persisted for up to 20 days under continuous s-µ*g* exposure. This indicates suppressing B or CD4 T-cell activity [170]. 

In another research study evaluating the immunological responses of individuals aged 21–55, mass cytometry and RCCS (5 rpm for 18 h) were utilized. The study also observed the cellular impacts of dysfunctional T cells, revealing heightened regulatory responses and reduced activity of NK effector cells, CD41 and CD81 [148]. A study has shown a notable decrease in the capacity of NK cells to eliminate target cells under simulated microgravity conditions. The study focused on various processes, such as cell death, receptor presence, and cytokine release in human NK cells under s-µ*g* conditions. The study noted that s-µ*g* exposure led to lower cell-killing ability and increased cell death (apoptosis) and tissue death (necrosis). Additionally, there was a noticeable decrease in interferon (IFN)-γ and perforin production, while granzyme-B production only decreased slightly. The specific receptors on NK cells, NKG2A and NKG2D, were specifically reduced by s-µ*g*, but the expression of NKp30 and NKp44 was unaffected [171]. During the BIO-4 mission on the ISS, an experiment was carried out to investigate the process of apoptosis in lymphocytes under microgravity conditions and its potential connection to 5-lipoxygenase (5-LOX). The findings indicated a considerable rise in apoptotic markers, such as DNA fragmentation and cleaved-poly (ADP-ribose) polymerase (PARP) protein expression, which were roughly three times higher than the controls on the ground. Moreover, the mRNA levels of apoptosis-related markers, including p53 and calpain, exhibited a three-fold and four-fold increase, respectively. Additionally, there was an early two-fold increase in 5-LOX activity [172].

A research study investigated cell structure changes and human macrophages’ energy production. The experiment, called “*CellBox-Primary Human Macrophages in Microgravity Environment*”, took place aboard the ISS. Proinflammatory M1 macrophages were exposed to µ*g* for 11 and 30 d. The findings revealed that the macrophages exhibited increased size and number in the space environment, indicating enhanced proliferation. Moreover, there was a decrease in ICAM-1 on day 11, a molecule that aids in recruiting immune cells, without significant differences in CD18 expression [173]. This implies a potential reduction in inflammatory responses in the space environment. Intriguingly, within the body, macrophage proliferation is a characteristic feature of Th2 immunity, serving as a mechanism to limit the influx of inflammatory cells into active areas by increasing the number of macrophage cells in those specific locations [174].

A recent study investigated how altered gravity affects human M1 macrophages using parabolic flight experiments. The researchers used chromatin immunoprecipitation DNA sequencing (ChIP-seq) to identify DNA-binding loci associated with critical gene regulatory components. The findings showed decreased binding sites for the epigenetic marker H3K4me3 under altered gravity, with a non-uniform distribution across the genome. Notably, these downregulated loci corresponded significantly to immunoregulatory genes. Additionally, analysis of RNA polymerase II binding indicated an altered transcriptional activity. This study highlights the complex relationship between gravitational changes, chromatin organization, and gene regulation in immune cells [175]. 

In an innovative research project, advanced next-generation sequencing (NGS) methods were used to explore the influence of s-µ*g* (HARV RCCS) on DNA methylation processes in human lymphoblastoid cells. The study revealed epigenomic alterations induced by brief exposure to s-µ*g*. Specifically, s-µ*g* exposure led to changes in the methylome. Approximately 60% of the differentially methylated regions (DMRs) showed reduced methylation levels, while around 92% of the differentially hydroxymethylated regions (DHMRs) exhibited increased hydroxymethylation. Furthermore, the study identified 370 transcripts with altered expression, impacting critical biological processes such as oxidative stress response, carbohydrate metabolism, and transcription regulation. Although a universal correlation between methylation/hydroxylation changes and gene expression was not universal, specific genes with distinct methylation patterns were identified [176].

Researchers isolated peripheral blood mononuclear cells from regular donors using human peripheral blood. These cells were cultured under normal gravity (1*g* in T flasks) and s-µ*g* (MACS; microgravity analog culture system developed by NASA) for 24 and 72 h [177]. Afterward, cell samples underwent gene array analysis using the Affymetrix HG_U95 array [177]. The information was then analyzed using a two-way analysis of variance. Researchers focused on different groups of genes related to the immune response, utilizing Spotfire and Unix software (https://pubmed.ncbi.nlm.nih.gov/19426312/, accessed on 6 September 2024). Notably, 78 genes were influenced by the type of gravity (1*g* or MACS), 176 genes were affected by the duration of exposure alone, and 44 genes showed an interaction effect between time and gravity type. Among these genes, specific examples were highlighted, including downregulation of T cell activation genes (e.g., DAG kinase, human serine/threonine kinase, and tyrosine kinase) and upregulation of others. The study sheds light on the impact of s-µ*g* on gene expression patterns relevant to immune function [177]. 

Related to T cell activation, NFAT, the transcription factor involved in the activation of T cells, which is activated by the Ca^2+^ influx/calcineurin pathway, was studied. It was found that NFAT binding to DNA was impaired owing to the downregulation of AP-1 in µ*g* conditions (clinorotation), but NFAT remained intact [178,179].

It is essential to characterize extra macrophage phenotypes, like M2 (tolerogenic/anti-inflammatory) groups, to thoroughly comprehend the intricacies of the immune response in the µ*g* environment. One notable finding is that in spaceflight and s-µ*g* environments, there is a general decrease in hematopoietic stem cell differentiation into M0 macrophage populations, as well as a decrease in macrophage differentiation/polarization phenotypes (M1 and M2) and ICAM-1 expression during the flight [74,180,181].

These results support the notion of restricted migration and activity of immune cells under µ*g*, which may lead to an overall reduction in immune function. However, given the current knowledge, an analysis of leukocyte migration behavior in the µ*g* environment of spaceflight has yet to be conducted. The NASA Twins study used single-cell RNA sequencing of lymphocyte-depleted blood collected during a 1-year trip (~340 days) and found a predominance of M1 during flight and M2 predominance afterward. This implies that post-flight tolerogenic and repair pathways are active, aiding muscle regeneration upon return to Earth [182]. Moreover, the study validated earlier findings that showed increased MCP-1 and IL-1RA expression post-flight (180-day mission) [183], indicating a potential increase in M2 populations. Furthermore, increased M1 predominance in astronauts during the spaceflight can indicate compensatory mechanisms in maintaining immunological homeostasis or may represent a phenotype associated with a specific stage of immune response kinetics.

Similarly, a recent study investigated the impact of a short-duration spaceflight on monocytes. The results revealed significant changes in monocyte phenotype and cytokine production profiles, emphasizing the mission-specific nature of these alterations. Noticeably, cytokine production profiles in monocytes from space-flown individuals were also found to be altered, potentially affecting immune responses during space missions. Of particular interest, the study highlighted a selective impact: specifically, IL-6 production showed a significant reduction following specific stimulation. Furthermore, the observed alterations in monocyte function were mission specific, influenced by factors such as mission duration, microgravity exposure, and individual variability. In summary, a short-duration spaceflight induces multifaceted changes in monocyte function, which could impact the overall immunocompetence of crew members [184].

Numerous studies have explored the impact of µ*g* on omics knowledge in macrophages/monocytes and other immune cells [185,186,187]. These investigations used animal models, mouse/rat cells, and human monocytic/macrophagic cell lines such as U937, J111, and FLG29.1 [166,188,189,190,191]. Although the last three cited cell lines are human leukemia cell lines and not within the scope of our review, it is worth noting that they showed a structural disorganization of the cytoskeleton, a decrease in actin, tubulin, and vimentin, as well as a reduction in the expression of the cell adhesion molecule ICAM-1 and CD18. A similar cytoskeletal response to µ*g* was noticed in human primary macrophages [192]. This study used the FLUMIAS (high-resolution fluorescence microscope for live-cell imaging) during the TEXUS-54 sounding rocket mission to study viable human macrophages. The scientists simultaneously marked the nucleus, cytoplasm, lysosomes, and actin cytoskeleton by employing various cellular dyes responsive to different laser wavelengths. The primary objective was to detect microgravity-induced changes, comparing these cellular structures to their counterparts under normal gravity conditions on Earth. Notably, the SiR-actin probe, designed for real-time cytoskeleton imaging, revealed significant shifts in cellular and nuclear shape, volume, and alterations in the cytoskeleton’s structure. These changes manifested rapidly, but for the flight, the cells adapted, highlighting their quick response to the low-gravity environment [148,192].

Moreover, CD14, a recognition receptor responsible for triggering the macrophage response, was impacted in these cell lines. Conversely, there was an observed upregulation of the HSP70 protein, a crucial stress protein that safeguards myeloid cells from apoptosis. Gene expression analysis indicated the downregulation of MAPK, PKC, and NFkB signaling pathways, upregulation of the p53 signaling pathway, and inhibition of nitric oxide production—a crucial signaling molecule of M1 macrophages. Increased secretion of cytokines (IL-1, IL-2, IL-8, TGF-β1, M-CSF, etc.) was noted in U937 cells cultured under microgravity, along with inhibition of the PMA signal transduction pathway [193] and inhibition of reactive oxygen species—molecules that modulate the immune response and reduce disease susceptibility.

Recent ISS research has shown that the *miRNA* expression was altered during spaceflight. One study revealed that the gene expression of *miR-21*, which plays a role in cell cycle progression and proliferation, was suppressed in primary human T cells stimulated by conA and anti-CD28 [189,194]. Microarray analysis also demonstrated changes in eighty-five genes, many of which are targets of and regulated by miR-21 during spaceflight. These findings suggest that gravity affects molecular activation in T cells by suppressing transcription factors and inhibiting noncoding RNA. 

Metabolic changes were recognized in human primary macrophages under µ*g* since it was observed that after just five minutes of µ*g* (TEXUS 54 sounding rocket mission), there was an increase in amino acid concentration, which then reversed after 11 days of continuous exposure (CellBox PRIME-ISS mission). Specific amino acids exhibited the most significant reactions to changes in gravity and were closely grouped. These findings suggested processes of protein degradation in microgravity. Furthermore, glucogenic and ketogenic amino acids were further degraded to produce glucose and ketoleucine. Remarkably, ketoleucine accumulated robustly in both short-term and long-term µ*g* but not in hyper-*g*. Overall, this study underscores the highly dynamic and adaptive metabolic changes induced by altered gravity, highlighting the potential contribution of metabolomic studies to our understanding of gravity’s effects on human cells [195].

Dang and colleagues studied the effects of s-µ*g* (RWV at 37 °C for 30 min) and radiation (heavy ion) on B cells. The researchers found that RWV exposure decreased radiation-induced cell survival and increased apoptosis in human B lymphoblast HMy2.CIR cells. In addition, it amplified heavy ion radiation-elicited intracellular ROS generation, which induced ROS-sensitive ERK/MKP-1/caspase-3 activation in HMy2.CIR cells. These results could be reversed by the antioxidants N-acetyl cysteine (NAC) and quercetin [196]. 

Other science teams studied the metabolism of endocannabinoids and related bioactive lipids under s- and r-µ*g* conditions. They observed an upregulation of 2-AG (2-arachidonoylglycerol) and AEA (N-arachidonoylethanolamine), while fatty acid amide hydrolase (FAAH) and cannabinoid receptors were downregulated. These substances are essential for the immune system response and bone restructuring [197,198,199].

Several studies on omics knowledge in macrophages and monocyte cells under µ*g* conditions were conducted in animal models or mouse/rat cells [165,200,201,202,203,204,205,206,207]. Furthermore, most cell culture studies examining the impact of µ*g* on human monocytic/macrophagic cells regarding the omics field were performed using the U937, THP1, J111, and FLG29.1 cell lines. The latter three are human leukemia cell lines. In spite that these three leukemia human macrophage cells are not within the scope of our review, it is worth mentioning that these human cell lines showed structural disorganization of the cytoskeleton with a decrease in actin, tubulin, and vimentin [43,166,173,208] in addition to a significant reduction in the cell adhesion molecule ICAM-1 and CD18 expression. CD14, a recognition receptor on the cell surface that triggers macrophage response, was also affected. Upregulation of the essential stress protein HSP70 was observed in these cell lines, which played a protective role against myeloid cell apoptosis [166]. Gene expression analysis showed downregulation of MAPK, PKC, and NFkB signaling pathways and upregulation of the p53 signaling pathway [193,209,210], in addition to inhibiting the production of nitric oxide, the vital signaling molecule of M1 macrophages. Increased secretion of cytokines (IL-1, IL-2, IL-8, TGF-β1, M-CSF, etc.) has been recognized in U937 cells cultured under microgravity, in addition to inhibition of reactive oxygen species, which is included in the modulation of immune response, and its reduction increases the disease susceptibility. Furthermore, a study on osteoclastogenesis using bone marrow macrophages FLG29.1 cultured in space, in comparison with ground controls, revealed higher expression levels of tartrate-resistant acid phosphatase and cathepsin K. Additionally, activation of the NFkB and MAPK signaling pathways was observed, indicating that microgravity could potentially promote osteoclastogenesis [211].

Further investigations using genetic, transcriptomic, proteomic, epigenomic, and metabolic data to identify benign human immune cell-associated markers are essential for understanding the immune cell changes and response in µ*g*. The current knowledge is summarized in Table 5. This underscores the need for additional research to elucidate the molecular mechanisms that drive immune responses during space missions and comprehend the connection between the affected genes in µ*g* conditions.

### 4.8. Musculoskeletal System 

During the first years of manned spaceflight, muscle atrophy due to disuse and loss of bone density due to lack of mechanical loading were among the prime health issues for astronauts. With the development of effective exercise devices such as the ARED, these problems are much less urgent nowadays. However, with plans for missions to the Moon, Mars, or even beyond, the quest for detailed knowledge of the influence of µ*g* on the human musculoskeletal system has not lost any of its relevance today. 

#### 4.8.1. Chondrocytes

Chondrocytes, the main cellular component of articular cartilage, are embedded in a strong yet elastic ECM that provides resistance against mechanical forces and friction for the joints [212]. Spaceflight and the resultant weightlessness have significant impacts on these chondrocytes. Research indicates that both lack or excess of gravitational force leads to alterations in the cytoskeleton of chondrocytes, affecting their mechanical properties and the ability to synthesize ECM components such as collagen and proteoglycans that may lead to degenerative joint diseases like osteoarthritis [213]. 

In a study by Ma et al., RNA sequencing and RT-qPCR-based transcriptome analysis were conducted on meniscus fibrochondrocytes from the inner avascular region of human menisci. These cells were exposed to s-µ*g* for seven days using a RCCS bioreactor. The analysis revealed a notable upregulation of key osteoarthritis markers, such as *COL10A1*, *MMP13*, and *SPP1*, along with downregulating chondrogenic markers, including *ACAN*, *COL1A2*, *COL2A1*, and *SOX9*. The study also highlighted sex-specific differences in gene expression patterns. Further investigation into sex-dependent molecular mechanisms using hub gene networks showed that upregulated DEGs in females on days 3 and 7 were associated with Wnt signaling (e.g., *CTSK*, *IRAK1*, *JUN*), VEGF signaling (e.g., *KDR*, *PIK3R1*), and NFκB signaling pathways (e.g., *BIRC3*, *CXCL12*). In contrast, males exhibited upregulation in ECM components and matrix remodeling enzymes [213]. Additionally, changes in the expression of genes involved in the cell cycle and apoptosis were identified in a second study, indicating that mechanical unloading might affect cell proliferation and survival in the meniscus tissue. The upregulation of osteoarthritis markers and downregulation of chondrogenic markers underscore the risk of joint degeneration in altered gravity [214].

Secretome analysis by Aissiou et al. revealed upregulated gene expression in proteins predicted to be secreted by bioengineered cartilage derived from induced hBMSCs exposed to r-µ*g* during parabolic flight (11 parabolas). Genes such as WNT7B and WNT9A, which are associated with decreased chondrogenic markers and chondrocyte hypertrophy, respectively, were only in female-derived samples significantly upregulated. Other upregulated secretome genes included *CLEC2B* and *RMGA*, which are implicated in protein and carbohydrate binding as well as cartilage homeostasis. RNA-seq and RT-qPCR analysis revealed sex-specific gene expression changes: genes such as *KTI12*, *HSPE-MOB4*, and *HSPA1A* were upregulated in female-derived cartilage, while *HSPA1B* increased in male-derived cartilage, indicating different stress responses. These findings underscore the sex-dependent transcriptomic responses to microgravity and the pivotal role of Hsp70 heat shock proteins in maintaining chondrocyte homeostasis [36].

Studies have shown significant effects on gene expression in chondrocytes when exposed to short-term and long-term microgravity [215,216]. During the 20th DLR parabolic flight campaign, Wehland et al. inferred through their experiments that when chondrocytes are isolated and subjected to altered gravity conditions, there is an upregulation of inflammatory markers *IL6* and *CXCL8* and a downregulation of growth factors *EGF*, *VEGF*, and *FGF17*. Significant changes in the expression of genes involved in cell signaling, ECM organization, and cellular stress were also observed. The altered gene expression impacted several biological pathways, including the PI3K-Akt signaling pathway and pathways involved in stress response and inflammation [217]. Similar upregulation and downregulation of these inflammatory markers and growth factors were observed by Steinwerth et al. while studying the structural and molecular changes in human chondrocytes under µ*g* conditions when chondrocytes were exposed to the RWV bioreactor. These changes also included disruptions in actin filaments and microtubule organization. The changes in the expression levels of *COL2A1* and *ACAN* show that the synthesis of key ECM components like collagen and proteoglycans was affected [218].

Along with molecular changes, morphological changes in chondrocytes can significantly impact their function and the overall health of cartilage. The chondrocyte cytoskeleton, including actin microfilaments, tubulin microtubules, and vimentin intermediate filaments, is sensitive to µ*g*, leading to altered cell shape and signaling. Aleshcheva et al. observed increased F-actin and *ACTB* mRNA upregulation in cells when subjected to µ*g* in an RPM, indicating significant cytoskeletal remodeling. The upregulation of *TGFB1* gene expression and increased TGF-β1 protein production were noticed, likely promoting ECM production under µ*g* conditions. TGF-β1 enhances structural protein production and influences cell behavior without affecting proliferation [215].

In another study by Aleshcheva et al., a parabolic flight experiment investigated the role of two specific genes, *BMP2* and *SOX9*, in mitigating the adverse effects of µ*g* on the cytoskeleton. The study found that upregulating *BMP-2* and *SOX9* genes could prevent microgravity-induced cytoskeletal alterations. The protective effects of *BMP-2* and *SOX9* are likely due to their roles in maintaining cytoskeletal integrity and promoting chondrocyte differentiation and function. These findings suggest that enhancing the expression of *BMP-2* and *SOX9* could be a therapeutic strategy to protect chondrocytes from the detrimental effects of µ*g* [216].

#### 4.8.2. Muscle Cells

As part of the Artificial Gravity Bed Rest with European Space Agency (AGBRESA) study, Ganse et al. exposed participants to 30 min continuous or intermittent centrifugation with 1*g* at the center of mass during a 60-day 6° head-down tilt bed rest study. By assessing circulating GDF-8/myostatin, slow skeletal muscle troponin T, prostaglandin E2, BDNF, GDNF, and C-terminal agrin fragment, as well as serum creatine kinase activity, they found that neither bed rest nor both modes of centrifugation had any impact on the neuromuscular secretome [100].

A combined multi-omics approach using transcriptomics, proteomics, and metabolomics analyses, a database comprising 4 human cell models, 13 different tissues (11 mice and 2 humans), 2 mouse strains (C57BL/6 and BALB/C), blood and urine metabolite levels from space missions ranging from 2006 to 2017, and transcriptional data from the NASA Twin Study found a significant enrichment in spaceflight-related regulations of mitochondrial processes, innate immunity, chronic inflammation, cell cycle, circadian rhythm, and olfactory functions. Interestingly, the authors also pointed out that altered mitochondrial function and mitochondrial stress are typical spaceflight phenotypes [154].

In the NASA twin study, serum levels of IL-6, CRP, IL-10, CCL2/MCP1, and IL-1ra showed a sharp spike at days 0 and 5 after return to Earth, while elevated in-flight markers associated with bone metabolism and early stages of muscle regeneration, such as serum MIP-1a, RANTES, IL-8, FGF, G-CSF, GM-CSF, IL-1, IL-1a, and TNF-α, as well as urinary CTX, DPD, and NTX, decreased upon landing [182].

A combined proteomic dataset of both single muscle fiber biopsies from 10 healthy volunteers undergoing 10 days of bed rest and muscle biopsies of two astronauts of equal sex and comparable age before and after their 6-month mission on the ISS was analyzed by two studies. Both conditions led to a downregulation of proteins involved in focal adhesions, fiber–matrix interaction, and insulin receptor stabilization, while reloading reverted these tendencies. Furthermore, the antioxidant response proteins only increased in slow but not in fast muscle fibers. An analysis of the astronaut samples alone revealed an upregulation of markers for neuromuscular damage [219]. Further in-depth analysis of the astronaut dataset showed a dramatic decrease in the expression of mitochondrial proteins located in all main compartments of the organelle, particularly the inner mitochondrial membrane and the matrix, which occurred during spaceflight. Irrespective of the amount and type of exercise, both astronauts lost 43% and 55% of their oxidative phosphorylation-related proteins, which quickly reverted upon return to Earth [220].

In another bed rest study, healthy male volunteers were assigned to three groups: 70-day head-down bed rest (HDBR) with placebo and no exercise (CON), HDBR with placebo and exercise (PEX), and HDBR with 100 mg testosterone enanthate (at days 7, 28, 35, 56, and 63) and exercise (TEX). Muscle biopsies were taken at days 1, 36, and 64. Isolated proteins were then analyzed for differential phosphorylation and abundance. The authors found 59 unique proteins significantly regulated due to BR and/or countermeasures. These proteins belonged to pathways such as calcium signaling, cellular effects of sildenafil (Viagra), epithelial adherens junction signaling, actin cytoskeleton signaling, and ILK signaling. Interestingly, a principal component analysis demonstrated that CON, PEX, and TEX induced distinct expression and phosphorylation patterns for each group. In CON samples, proteins related to muscle contraction (MYH2, ACTG2, TPM2, TRIM72, ANKRD2) were found, whereas PEX mainly affected proteins for the cellular organization in muscle (ACTA1, TPM2, TPM1, KRT9, DES), and TEX proteins that mediate mesenchymal migration (ACTA1, ACTG2, ACTC1). Furthermore, several biomarkers for the susceptibility to HDBR and the effectiveness of countermeasures were identified [221].

Several other studies focused on exercise’s effects. McFarland et al. showed that during a 5-week-long 6° head-down bed rest, 1352 differentially expressed genes were found in test subjects without exercise but only 132 in subjects with exercise intervention. Among those genes in the non-exercise group, ligands to receptors on dorsal root ganglion neurons were enriched. They propose that exercise prevents the sensitization of dorsal root ganglion neurons [92].

A quantitative 2D differential gel-electrophoresis analysis of vastus lateralis (VL) and calf soleus (SOL) biopsies of subjects undergoing 55-day bed rest during the Berlin Bed Rest Study 1 with or without vibration exercise countermeasures showed an increase in myosin heavy chain type I and a decrease in type II in both muscle types that could be reverted with exercise. Furthermore, bed rest alone led to a downregulation of proteins involved in aerobic metabolism. This was reversed by exercise, albeit more in VL than SOL. In addition, exercise also upregulated proteins involved in anaerobic glycolysis [222].

Using the results from [222], Teodori et al. used an in silico approach to predict possible microRNAs (miRNAs) that may be involved in the observed deregulation of proteins. Overall, they found the miRNAs let-7a-5p and miR-125b-5p as potential candidates involved in the overexpression of genes in SOL and VL, and miR-1-3p, miR-125b-5p, and miR-1-3p as well as miR-95-5p as potential candidates involved in downregulated genes in SOL and VL [223].

In the subsequent 2nd Berlin bed rest study, 12 subjects underwent 60 days of −6° head-down tilt bed rest with either no exercise (control), resistive exercise (RE), or resistive exercise with whole-body vibrations (REV). Samples from the soleus muscle before and after the study were taken and analyzed for differential gene expression employing the microarray technique and validated by 2D differential gel electrophoresis. While bed rest alone resulted in 235 differentially expressed genes, RE only resulted in 206 differentially expressed genes, and REV reduced this number to just 51. Most of these genes belonged to metabolic pathways such as glycolysis, oxidative phosphorylation, tricarboxylic acid (TCA) cycle, lipid metabolism, as well as to myofibers and muscle development. Interestingly, the three groups of differentially expressed genes only shared little overlap (56 in control and RE, 4 in control and REV, and 4 in RE and REV), with just one gene being differentially expressed in all three groups (hexokinase 2, *HK2*) [224]. 

#### 4.8.3. Bone Cells 

During the VITA mission of the Italian Space Agency, Gambacurta et al. studied proteomic and epigenetic changes in human blood-derived stem cells during osteoblastic differentiation induced by rapamycin under µ*g* conditions. Cells cultured on the ISS were characterized by a decreased protein expression of Sox2, Oct3/4, Nanog, and E-cadherin accompanied by an expression pattern of the transcription factors Otx2, Snail, GATA4, and Sox17. They exhibited an earlier activation of the differentiation process towards the osteogenic lineage. Furthermore, the authors could also demonstrate that the residues H3K4me3, H3K27me2/3, H3K79me2/3, and H3K9me2/3 of histone H3 were involved in the epigenetic regulation of osteogenic differentiation [225].

Li et al. exposed osteogenically induced human bone marrow mesenchymal stem cells to s-µ*g* on the RPM for 2, 7, and 14 days and identified 837, 399, and 894 differentially expressed genes, respectively. After two days, a significant downregulation of genes belonging to biological processes such as cell cycle, mitosis, cell division, or nuclear division was found, hinting towards a stunted proliferation, while at day 7, biological processes such as regulation of ossification, vasculature development, regulation of multicellular organismal development, or positive regulation of developmental processes were enriched with upregulation of genes involved in the PPAR signaling pathway, and suppression of genes from the calcium signaling pathway, pancreatic secretion, or GnRH signaling pathways, which indicates a suppressed osteogenic differentiation. After 14 days, the enriched biological processes were mainly system development and single organism developmental processes, single multicellular organismal processes and related, with the most prominent pathways of tumorigenesis [226].

In a further in vitro experiment employing the RPM, Zhivodernikov et al. exposed intact and osteocommitted human mesenchymal stromal cells to 10 days of s-µ*g*. The expression of 84 genes associated with adhesion and matrix production was analyzed. It was found that RPM exposure led to similar effects in osteocommitted and intact cells, but the differences were more pronounced in the former (*COL11A1*, *CTNNB1*, *HAS1*, *ITGA3*, *ITGB1*, *LAMA3*, *MMP1*, *MMP11*, and *TNC* vs. *COL11A1*, *CTNND1*, *TIMP3*, and *TNC* with more than 1.5-fold difference and *p* ≤ 0.05) [227].

Cells from the human osteoblast-like line MG-63 were subjected to diamagnetic levitation in an apparent gravity of 0, 1, and 2*g* vs. control. A transcriptomic microarray analysis with subsequent qPCR validation focusing on cytoskeleton-related genes revealed a group of 13 mechanosensitive genes in a comparison of all conditions (*ADD3*, *COTL1*, *FLNA*, *SORBS3*, *CDC42BPB*, *TMOD3*, *TLN1*, *SPTBN1*, *SVIL*, *WASF2*, *WIPF1*, *PLEC*, and *PXN*), of which only *TLN1*, *SPTBN1*, *COTL1*, and *WASF2* were significantly regulated in µ*g* vs. control [228].

Bradamante et al. cultured human bone marrow stem cells treated with the physiological osteoinducer 1,25-dihydroxy vitamin D for 14 days in space on board the Soyuz capsule and the ISS. A combined microarray and RNA-seq gene expression analysis revealed 391 unique genes identified only by RNA-seq, 153 unique genes identified only by microarray analysis, and 89 genes identified by both techniques. The shared genes belonged to pathways such as notch-mediated HES/HEY network, notch signaling pathway, beta 1 integrin cell surface interactions, integrin family interaction, epithelial-to-mesenchymal transition, and VEGF or VEGFR signaling network. These genes unique to RNA-seq belonged to beta_1_-integrin cell surface interactions, integrin family cell surface interactions, ErbB receptor signaling network, GMCSF-mediated network, IL-5-mediated signaling events, or IL3-mediated signaling events, while the genes detected only by microarray analysis belonged to epithelial-to-mesenchymal transition, beta_3_-integrin cell surface interaction, developmental biology, axon guidance, hemostasis, or integrin family cell surface interactions. An additional exosomal microRNA analysis in spent media revealed candidates in MSC mechanosensing. The authors propose that from those genes and miRNAs detected in this story, PPRy, *SOX9*, *MEK/ERK*, *LIP*, *Msx2*, *RB1*, *S100a16*, *Mir29A*, *SFRP-4*, *Mir31*, *MirLetA2*, *RUNX2*, *COL1A*, *BGLAP*, *RANKL*, *ALP*, *CYP27A*, and *TAZ* play a central role in mechanosensing, mechanotransduction, and differentiation of MSC in µ*g* [229]. Using the GSE100930 dataset from the Gene Expression Omnibus (GEO) database (generated from data from [229]), Wei et al. conducted a bioinformatic analysis of long non-coding RNA expression profiles in bone marrow mesenchymal stem cells under µ*g*. They found 2212 significantly regulated mRNAs (975 upregulated and 1237 downregulated) and 22 differentially expressed lncRNAs (12 downregulated and 10 upregulated). The construction of a coding–non-coding gene co-expression (CNC) network revealed that the lncRNAs GSN-AS1, EPB41L4A-AS1, TP53TG1, MIR155HG, RNF185-AS1, and SNHG12 were hubs within this network. Furthermore, by using the expression patterns of the top 30 differentially expressed mRNAs to interrogate the CMap database, the compounds with the highest negative enrichment scores were scoulerine, kinetin riboside, and dexanabinol, which the authors propose as potential therapeutic agents against disuse osteoporosis [230].

A very similar approach, focusing more on mRNAs and miRNA–mRNA interactions, was chosen by Zhang et al. However, they opted to include datasets from different species (mice: GSE1367, GSE 4658, GSE78980, and SRP276872; humans: GSE100930, GSE 100932, and GSE114117) and different culture conditions (RPM, RWV, RCCS, spaceflight). They found that the strong variety of the data made it difficult to reach consistent results across all included studies. However, in the human samples, they identified 11 hub genes (*IL6*, *MAPK3*, *JUN*, *CXCL8*, *IL18*, *FGF2*, *IGF1*, *PTGS2*, *CCL2*, *FOS*, and *ICAM1*) in the data from [229]. *CCL2*, *ICAM1*, *IGF1*, *miR-101-3p*, and *miR-451a* were also identified as significantly regulated under clinorotation, with *ICAM1* and *miR-451a* playing a central role in osteogenesis under s-µ*g* [231].

In a proteomic study by Montagna et al., human bone marrow stromal cells were cultured in osteogenic medium on the RPM for 8 and 28 days. Overall, they found 481 differentially abundant protein groups (DAPGs). Thirteen cellular component categories were significantly enriched in a GO annotation overrepresentation analysis, with 49 DAPGs belonging to the ECM, 28 to the mitochondrial matrix, 47 to the cell–substrate junction, and 30 to the actin cytoskeleton. Furthermore, the DAPGs were also categorized into enriched major GO biological processes: cell fate (comprising proliferation, differentiation, adhesion, signaling, and death) and cell metabolism (comprising carbohydrates, lipids, proteins, transport, and nucleic acids). The authors state that their data indicate that humans’ primary BSMCs undergo different phases under s-µ*g*. The first phase until day 8 involves cytoskeleton rearrangement and reduced osteogenic differentiation. In the later phase, the cells display high plasticity as a sign of their adaptation to the culture conditions. ECM mineralization occurs, but the cellular metabolism remains changed [232].

To assess the effect of s-µ*g* on human primary osteoblasts, Michaletti et al. exposed these cells to the RPM for 110 h and analyzed them subsequently for proteomic and metabolomic changes. Approximately 813 and 978 proteins were identified in 1*g* and RPM samples, respectively, with 562 of them present in both groups. Interestingly, over 50% of all proteins sensitive to culturing on the RPM were related to the mitochondrion and mitochondrial processes such as mitochondrial electron transport or mitochondrial protein homeostasis. Focusing on proteins from the respiratory chain, the glycolytic metabolism, and antioxidant enzymes, the authors identified 28 significantly regulated mitochondrial proteins belonging to the enriched molecular function items glycolysis, Krebs cycle, pentose phosphate pathway (PPP), ubiquinol-cytochrome-c reductase activity, cytochrome c oxidase activity, and antioxidant activity. The metabolomic profiling of the cells confirmed a significant reduction in glycolysis, Krebs cycle, PPP, the glycerol-phosphate shuttle, and the malate-aspartate shuttle on the RPM. Overall, the authors conclude that culture on the RPM may inhibit bone cell functions, thus disturbing the cell’s energy balance [233].

Overall, these studies show that the reaction of bone and muscle cells to both r- or s-µ*g* is a complex process that still needs thorough, in-depth analyses. Omics can be a valuable tool for this purpose, as they might help to filter more subtle effects from vast datasets and can be used to re-analyze and refine older data findings. Table 6 summarizes the most important papers available in this research field.

### 4.9. Endocrine System 

#### 4.9.1. Prostate Cells 

Several manufacturers can purchase primary human prostate epithelial cells. Unfortunately, no studies have been published investigating this cell type under µ*g* conditions. Most available publications focused on prostate cancer cells [234,235,236]. Only a few studies investigated prostate fibroblasts under s-µ*g* conditions.

Ingram et al. exposed various cell types to the NASA-HARV (high aspect ratio vessel) bioreactor. Among others, they also focused on normal prostate fibroblasts [237]. On day 4, normal prostate fibroblasts (NPF 209) grew in compact multicellular spheroids approximately 0.5 mm in diameter and stained intensely throughout for collagen IV [237]. Coculture experiments of fibroblast spheroids with PC3 prostate cancer cells showed enhanced tenascin expression by the fibroblasts underlying the adherent prostate epithelial cells. An invasion of the fibroblast spheroids by the malignant PC3 epithelium was evident [237].

A second study focused on the effects of androgen on growth and PSA expression by LNCaP prostate cancer cells cultured alone or mixed with human prostate fibroblasts [238]. The coculture experiment revealed that the cells exposed to s-µ*g* conditions responded to the inductive growth and differentiation signals from dihydrotestosterone, similar to the in vivo situation [238].

One long-term study investigated ex vivo intact prostatic tissue obtained from transurethral prostatectomy or biopsy on the RWV for 28 days [239]. Intermediate filaments like cytokeratins or vimentin and TGF-P2 receptors and ligands were preserved throughout the culture period. Prostatic acid phosphatase (PAP) was slightly decreased. Prostatic-specific antigen (PSA) and its transcript were downregulated. 

Taken together, only a few older publications report some omics data of healthy cells deriving from the prostate.

#### 4.9.2. Thyroid Cells 

Prolonged exposure to the orbit microgravity environment can disrupt the thyroid gland’s function in vivo. It showed various changes, such as follicles with larger thyrocytes and elevated levels of cAMP, thyrotropin receptors (TSHR), and caveolin-1 [240,241].

Normal human HTU-5 thyroid cells studied on a 3D clinostat for 24 h demonstrated moderate signs of apoptosis as shown by electron microscopy, caspase-3 activation, elevation of Fas protein, and an increase in 85 kDa apoptosis-related cleavage fragments resulting from enhanced poly(ADP-ribose) polymerase activity [242].

FRTL-5 thyroid cells were exposed to µ*g* during the TEXUS-44 sounding rocket mission (launched 7 February 2008, from Kiruna, Sweden) [243]. Under r-µ*g* conditions, the thyrocytes did not respond to TSH [243]. The authors showed an irregular cell shape with condensed chromatin, a modification of the cell membrane, and the shedding of the TSH receptor in the supernatant. Moreover, the authors reported an elevation of sphingomyelin-synthase and Bax proteins [243]. 

Albi et al. investigated HBME-1, MIB-1, CK19, and galectin-3 proteins in the thyroid of mice remaining in space for 90 days on the ISS [241]. MIB-1 and CK19 were unchanged, but HBME-1 and galectin-3 were overexpressed in space. The authors showed that galectin-3 had left the thyrocytes and was detectable in the colloid. This finding might be due to a membrane remodeling of the thyroid cells in r-µ*g* [241].

Normal Nthy-ori 3-1 thyroid cells and the low-differentiated follicular thyroid cancer cells FTC-133 were exposed to an RPM for 7 and 14 days [244]. The FTC-133 cells formed larger MCS and a greater number of them than normal cells. The gene expression of *IL6*, *IL7*, *IL8*, *IL17*, *OPN*, *NGAL*, *VEGFA*, *ACTB*, *TUBB*, *PFN1*, *CPNE1*, *TGM2*, *CD44*, *FLT1*, *FLK1*, *PKB*, *PKC*, *ERK1/2*, *Casp9*, *Col1A1,* and the release of the following proteins (IL-6, IL-7, IL-8, IL-17, OPN, NGAL, VEGFA) was analyzed. Impressive differences between benign Nthy-ori 3-1 and malignant FTC-133 cells were found regarding the expression of *NGAL*, *VEGFA*, *SPP1*, *IL6*, and *IL17* mRNAs and the secretion of VEGFA, IL-17, and IL-6. This data proposes that gravisensitive growth factors or angiogenesis markers play a role in 3D growth and metastasis of thyroid cells exposed to s-µ*g* conditions [244].

Healthy thyrocytes of the human follicular epithelial thyroid cell line Nthy-ori 3-1 exposed to the RPM showed a significant upregulation of *IL6*, *CXCL8*, and *CCL2* mRNAs after 4 h [245]. After a 24 h RPM exposure, significantly reduced IL-6, CXCL8, FN1, ITGB1, LAMA1, CCL2, and TLN1 gene expression was measured in MCS compared to 1*g* controls [245]. After a 72 h RPM exposure, secretory changes were detected. These thyrocytes’ IL-6, IL-8, and TIMP-1 secretion rates were significantly increased. This study demonstrated that normal thyroid cells formed MCS within one day, and cytokines seem to be involved in this process [245].

A new investigation focused on the impact of dexamethasone (DEX) on benign Nthy-ori 3-1 cells in comparison to malignant thyroid cancer cells [246]. DEX did not influence the spheroid formation of Nthy-ori 3-1 thyrocytes and recurrent ML-1 follicular thyroid cancer cells. These data contrast with low-differentiated FTC-133 cells. The authors demonstrated that Nthy-ori 3-1 and ML-1 cells upregulate the anti-adhesion protein mucin-1 when cultured on the RPM, presumably as a protection mechanism against mechanical stress [246]. Moreover, an 8-fold increase in fibronectin in DEX-treated Nthy-ori 3-1 cells was measured. In Nthy-ori 3-1 cells, laminin was slightly increased in the DEX-treated RPM group. While the amount of MMP-9 was not affected by either RPM or DEX influence, MMP-2 was decreased after DEX administration in Nthy-ori 3-1 cells. Nthy-ori 3-1 cells exhibited generally lower levels of MMP-2 and MMP-9 than the malignant cell types. Nthy-ori 3-1 cells also activated p38 stress pathways on the RPM, but to a lesser extent and without sensitivity to DEX [246].

Taken together, only a few publications focusing on omics investigations with normal thyroid gland cells are available today (Table 7).

#### 4.9.3. Pancreas Cells 

The pancreas is a retroperitoneal organ with exocrine and endocrine functions. Histological investigation of the pancreas reveals serous ductal acinar exocrine cells. The gland shows a tubuloalveolar arrangement with the islets of Langerhans and the endocrine cells. The PubMed search of “Pancreatic cells and microgravity” gave 31 hints, and finally, the “human pancreatic cells and microgravity” search delivered 12 papers (5 July 2024). Only one publication (selection criteria: English language, human cells, and only original articles) met the criteria and could be included in this review.

Pancreatic cells were exposed to s-µ*g* and mainly used for tissue engineering [247]. Islet cells derived from patients with persistent hyperinsulinemic hypoglycemia of infancy (PHHI) were cultured in the HARV. This technology made it possible to reactivate insulin, glucagon, somatostatin, and GAD expression in PHHI-derived cells that had previously stopped expressing these markers [248].

In summary, most of the available literature reported studies with animal cells, and only one science team used human cells and studied them under µ*g* conditions (Table 7).

### 4.10. Digestive System 

The effects of gravitational changes on the digestive system are complex. Studies on human cells or humans are very limited, but animal studies have already provided evidence of the complex expected effects of µ*g* on the human digestive system (Table 8; summarized in [249]).

#### 4.10.1. Stomach

A comparative proteomic analysis of human gastric mucosal cell cultures under s-µ*g* (HARV) conditions was first performed by Lu et al. [250]. The authors identified 394 proteins differentially regulated after 3 days and 542 proteins differentially regulated by s-µ*g* after 7 days. GO and KEGG pathway enrichment analyses showed that after three days, proteins related to ribosomes, the response to ER, and the NFκB signaling pathway were significantly upregulated, suggesting that the cells may be under stress and exhibit inflammation. After 7 days on the HARV, the oxidative stress associated with it was significantly upregulated. At the same time, an enrichment of the NFκB signaling pathway and a weakening of cell–cell junctions were observed. These results indicated that gastric mucosal cells were exposed to oxidative stress, inflammation, and weakening of cell–cell junctions after a short exposure to s-µ*g* (HARV) [250].

#### 4.10.2. Intestinal Tract

The influence of s-µ*g* (RWV) on the phenomics of human intestinal epithelial cells was described early on by Goodwin et al. [251] when normal small intestinal epithelium and mesenchymal cells were co-cultivated on Cytodex-3 microcarrier beads exposed to the RWV for 30–40 days. The epithelial models displayed apical brush borders, differentiated epithelial cells, cellular polarity, extracellular matrix, and basal lamina [251]. The effects of s-µ*g* (RWV) on epithelial barrier function were later analyzed by Alvarez et al. [252]. Intestinal epithelial cells exposed to the RWV for 18 days showed delayed localization of the tight junction proteins occludin and ZO-1 at the apical junction. The authors also observed that s-µ*g* induces a fundamental and persistent susceptibility to epithelial barrier disruption after leaving the s-µ*g* environment. This would have implications for the gastrointestinal homeostasis of astronauts in space and their ability to withstand the effects of substances that impair intestinal epithelial barrier function upon return to Earth [252].

#### 4.10.3. Liver

Khaoustrov et al. [253,254] found that the s-µ*g* environment is conducive to maintaining long-term cultures of functional hepatocytes. They cultured primary human liver cells on an RCCS for 60 days. The cells formed clusters of cohesive hepatocytes surrounded by complex stromal structures, reticulin fibers, and bile canaliculi with multiple microvilli and tight junctions. Albumin mRNA was expressed throughout the culture. The inhibited proliferation observed in Chang liver cells exposed to s-µ*g* (3D clinostat) could be explained by cell cycle arrest due to a downregulation of several cell cycle-related proteins, such as cyclin A1 and A2, cyclin D1, and cyclin-dependent kinase 6 after 72 h. s-µ*g*-exposed cells exhibited downregulation of α-tubulin-3 and β-actin, resulting in cytoskeletal reorganization [255]. s-µ*g* (RCCS) culture also promoted the 3D growth of human hepatic HepG2 cells and human biliary tree stem/progenitor cells (hBTSCs) [256]. A significant increase in the expression of stem cell genes was found in s-µ*g* (RCCS) in hBTSCs. The expression of hepatocyte lineage markers was significantly lower in hormonally differentiated hBTSCs. This finding indicates a limited ability of hBTSCs to differentiate into mature hepatocytes under s-µ*g* conditions. HepG2 cells showed a lower transcription of *CYP3A4*. Exo-metabolome NMR analysis showed that hBTSC and HepG2 cell cultures on the RCCS consumed more glucose and less glutamate. hBTSC cell culture supernatants contained more fermentation and ketogenesis products. HepG2 cells showed a higher consumption of amino acids and a higher release of keto acids and formiates. Based on these findings, the authors suggested that s-µ*g* could be useful for developing liver constructs from hBTSCs [256]. 

Fujisawa et al. [257] found evidence that liver fibrosis appears limited in a monoculture of activated primary human hepatic stellate cells under s-µ*g* (3D clinostat). Serial analysis of gene expression revealed the presence of sirtuin, EIF2 signaling, Hippo signaling, and epithelial adherens junction signaling. Upstream regulators such as Smad3, NFκB, and fibronectin were activated, and cell-permeable inhibitors such as Ly294002 and U0126 were inhibited. Immunohistochemical studies to evaluate cytoskeletal changes showed that more β-actin was localized in the cortical layer on the 3D clinostat [257].

**Table 8:** Omics studies on the digestive system under microgravity conditions.

Cell line/Tissue/Sample Type	Omics	µ*g*	Device/Platforms	Refs.
GES-1 gastric mucosal cells	Proteomics: 394 (3 d)/542 (7 d) proteins differentially regulated in s-µ*g*; oxidative stress; inflammation; cell connections	s-µ*g*	HARV, 3 d and 7 d	[250]
T-29.cl19a intestinal epithelial cells	Proteomics: delayed localization of occludin and ZO-1	s-µ*g*	RWV, 18 d	[252]
Primary human liver cells	Transcriptomics: albumin mRNA was expressed throughout the 60 d culture; coculture of hepatocytes with HMVECs stimulated albumin mRNA expression	s-µ*g*	RCCS, 12 d, and 60 d	[253,254]
Chang liver cells	Proteomics: downregulation of cyclins A1 A2, D1, cyclin-dependent kinase 6, α-tubulin-3 and β-actin	s-µ*g*	3D clinostat, 72 h	[255]
Human biliary tree stem/progenitor cells	Transcriptomics: increase in the expression of stem cell genes (*OCT4*, *Nanog*, *EpCAM*, *LGR5*, *SOX9*, *SOX17*, *PDX1*) Metabolomics: more fermentation (lactate, acetate) and ketogenesis (B-hydroxybutyrate) products.	s-µ*g*	RCCS, 14 d	[256]
HepG2 cells	Transcriptomics: downregulation of *CYP3A4* Metabolomics: higher consumption of amino acids, higher release of keto acids (3-methyl-2-oxovalerate, 2-oxo-4-methyl-valerate) and formiates			
Primary human hepatic stellate cells	Transcriptomics: upregulation of sirtuin, EIF2 signaling, Hippo signaling, and epithelial adherens junction signaling. Metabolomics: ROS upregulation	s-µ*g*	3D clinostat, 10 d	[257]

### 4.11. Skin

The effect of µ*g* on human skin is of great importance, as astronauts often complain about skin rash after long-term spaceflights, and no targeted therapy has been developed so far [258]. Sometimes, this pathology occurs due to herpes simplex type 1 reactivation [259], presumably due to a suppressed immune system [260], but sometimes the reason is still unknown. Wound healing, on the other hand, is also impaired under µ*g* microgravity, both under s- [261] and r-µ*g* conditions [262]. Therefore, it is worthwhile to discuss the effects of µ*g* on the major cell components of the skin, i.e., keratinocytes, dermal fibroblasts, and dermal microvascular endothelial cells.

#### 4.11.1. Keratinocytes 

Keratinocytes show intensive reactions to mechanical forces. This has been shown by the “addition” of mechanical forces in several bioreactor models. For instance, mechanotransduction by β1-integrin in a static stretch device was a crucial factor in keratinocyte migration [263]. Cyclic strain, furthermore, alters the expression and distribution of cell junction proteins in this cell type [264]. Therefore, one might conceive that “addition” and “reduction” of mechanical forces may alter omics in these mechanosensitive cells. So far, all data about the impact of µ*g* on keratinocytes has been obtained by simulation devices; no data is available from spaceflight experiments to date. s-µ*g* leads to phenotype changes in keratinocytes. 

Another experiment showed altered migration abilities of human keratinocytes exposed to an RPM and the formation of organoid-like structures in coculture with dermal fibroblasts [265]. Interestingly, gravitational forces also seem related to circadian rhythms in human keratinocytes [266]. The circadian rhythm gene *Bmal1*, after synchronization by DEX administration, showed stronger oscillations in expression under s-μ*g* than under 1*g* conditions, while a similar effect could also be observed for the repressor gene *Rev-erbα*. These experiments found no influence on either proliferation or apoptosis by s-μ*g* after 60 h. 

The coculture composed of human keratinocytes, human skin fibroblasts, and human umbilical vein endothelial cells (HUVECs) as substitutes for epithelial cells in Matrigel revealed a decrease in the diameter of the keratinocyte and the fibroblast layer under s-μ*g* (2D clinostat) conditions, which was interpreted as a mechanism similar to skin thinning during spaceflight [260]. 

Altered migration abilities of human keratinocytes under s-µ*g* (RPM) were also observed in another study, where the cells showed enhanced migration in a scratch test assay [265]. Furthermore, the keratinocytes revealed changes in cytoskeleton arrangement, with an alignment of action filaments more in the shape of cortical bundles. In contrast, actin filaments were aligned more longitudinally under 1*g* conditions. Changes in the invasive and migratory properties of the keratinocytes under s-μ*g* were interpreted as epithelial-to-mesenchymal transition (EMT). Changes in the expression of some markers were in accordance with this presumption. Hence, E-cadherin as an epithelial marker was decreased, while Snail1, α-SMA, vimentin, and fascin were increased on the protein level. On the mRNA level, *SNAIL1*, *SNAIL2*, *ZEB2*, and N-cadherin mRNA expression were increased compared to 1*g* after 24 h. Matrix metalloproteinases were activated by further changes towards a more invasive phenotype or a phenotype that gets prepared to perform wound healing. The gene expression of *MMP1*, *MMP2*, and *MMP9* was upregulated under s-μ*g*, while the expression of *MMP3* was downregulated. Proliferation and apoptosis, however, remained unaffected [265]. 

Another study investigated keratinocytes exposed to s-μ*g* created by an RWV device for up to 10 days [267], followed by recovery culturing for 15, 50, and 60 days in normal gravity. The gene expression profile showed 162 DEGs, 32 of which were “center genes” consistently affected in the time course experiments. Eleven center genes belonging to the integrated stress response pathways were decreased. In addition, seven center genes, which are all metallothionein MT-I and MT-II isoforms, were upregulated. In addition, HLA-G was significantly upregulated during the recovery phase. Overall, more than 80% of the DEGs from the shorter exposures (≤4 d) recovered in 15 d; for longer (≥9 d) exposures, more than 50 days were needed to recover to the impact level of shorter exposures. The data indicated that shorter RWV exposure times might lead to a faster and more complete recovery from the µ*g* influence [267].

#### 4.11.2. Dermal Fibroblasts 

Likewise, as a cell type related to keratinocytes in location and function, dermal fibroblasts are also affected by the impact of µ*g*. Dermal fibroblasts transferred to space during the Spacelab D2-mission in 1993 showed an increased collagen synthesis, with a decrease during hyper-*g* forces that occurred on the way to space [268]. On the other hand, the accumulation of dermal fibroblasts and the wound area was attenuated in a rat hindlimb unloading model to simulate µ*g* [261]. On the in vitro level in cell cultures, a scratch test revealed a decreased migration of dermal fibroblasts cultured on a 2D clinostat compared to 1*g* conditions. Mechanotransduction seems to play a role in this process. The mechanotransduction factor yes-associated protein (YAP) is involved in these processes, and its function is described elsewhere [269]. In a 3D clinostat for μ*g* simulation, dermal fibroblasts showed decreased proliferation and an increase in apoptosis, together with marks of elevated oxidative stress [266]. Concerning the cell cycle, more dermal fibroblasts were found in the G2/M phase and less in the S phase compared to the control. 

Dermal fibroblasts cultured under s-μ*g* provided by an RPM (24 h) showed a decreased migration in a scratch assay and decreased invasion in matrigel [266]. Correspondingly, the activity of matrix metalloproteinase 9 (MMP-9) was decreased under s-μ*g*, whereas the activity of MMP-2 remained unaltered [266]. The myofibroblastic markers α-SMA and cofilin were significantly downregulated under s-μ*g*, as was the expression of the actin-bundling protein fascin. 

RPM exposure impairs fibroblast conversion into myofibroblasts and inhibits their migratory properties. Fibroblast and keratinocyte cocultures showed phenotypic changes associated with the downregulation of α-SMA that translocates in the nucleoplasm. Stress associated with µ*g* resulted in oxidative damage. This data proposes establishing antioxidant strategies as countermeasures to the μ*g*-induced disruptive effects on fibroblasts [266].

Transcriptional and translational changes in juvenile human dermal fibroblasts exposed to an RPM were demonstrated by another study [270]. RPM exposure induced changes in morphology and alterations in the cytoskeleton, ECM, focal adhesion, and growth factors. RPM-exposed cells grew adherently and as multicellular spheroids (MCS). For both cell populations a differential regulation of fibronectin (*FN1*), laminin (*LAMA3*), collagen-IV (*COL4A5*), aggrecan (*ACAN*), osteopontin (*SPP1*), TIMP-1 (*TIMP1*), integrin-β1 (*ITGB1*), caveolin-1 (*CAV1*), E-cadherin (*CDH1*), talin-1 (*TLN1*), vimentin (*VIM*), TGF-β1 (*TGFB1*), IL-8 (*CXCL8*), MCP-1 (*CCL2*), MMP-1 (*MMP1*), and MMP-14 (*MMP14*) was measured. In silico analyses revealed interacting proteins involved in MCS growth via signals mediated by integrin-β1, E-cadherin, caveolin-1, and talin-1 [270]. 

Similar effects were observed in a study by Radstake et al., where dermal fibroblasts were exposed to either s-µ*g* (RPM) or hyper-*g* (large diameter centrifuge (LDC)) [271]. As shown in the previous study, cell migration decreased under s-µ*g*, but not significantly; the number of focal adhesions was reduced, actin structure was altered, and stress fibers were reduced. Remarkably, cortisol as a stress hormone considerably enhanced the effects of both hyper-*g* and s-µ*g* on the dermal fibroblasts regarding the production of inflammatory cytokines and ECM proteins. In vitro and in vivo, skin with dermal fibroblasts as its main component shows changes under µ*g*. During a mission on the ISS, mice were kept in r-µ*g* for 21 to 24 days [272]. Under these conditions, the skin showed signs of weakening, such as decreased procollagen fibers and increased collagen-3 content.

#### 4.11.3. Dermal Microvascular Endothelial Cells 

Dermal microvascular endothelial cells (DMECs) are relatively rare and difficult to grow, so studies about these cells under µ*g* do not exist in abundance. Extensive cytoskeleton remodeling was observed in a study using RWV devices to simulate µ*g* in human DMECs [273]. Disruption of the cytoskeleton and loss of stress fibers occurred. TRPM7 was suggested as a possible mechanosensor, as this cation channel contains an α-kinase domain that is known to be mechanosensitive [274]. It was downregulated under s-μ*g* in these experiments. The stress response seemed to occur distinctly, as indicated by the activation of stress proteins such as HSP70, TXNIP, P-HSP27, and HSP27. In more detail, further research is required to study the effects of s- and r-µ*g* on this integral compound of the skin. The available publications are summarized in Table 9.

## 5. Summary

This review article explores the µ*g* effects on various specialized cells and stem cells, with a focus on omics studies conducted in both, r-μ*g* platforms (ISS, sounding rockets, parabolic flights, drop towers) and s-μ*g* using ground-based devices (e.g., RPM, RWV, 2D and 3D clinostat). We discuss significant cellular structural and functional changes caused by µg, which can lead to health issues for humans in space. 

In summary, cells exposed to µ*g* conditions showed many common changes independently of the cell type. R-μ*g* or s-μ*g* induced significant morphological and structural alterations on stem cells, primary cells, and specialized cells, as well as cancer cells [43]. Common responses to µ*g* exposure include an increase in apoptosis [68,75,79,128,172,190,218], changes in the cytoskeleton [15,75,82,135,137,173,192,208,215], differentiation [76,82,106,111,112,225,226,229], proliferation [77,78,79,82,83,84,89], migration [82], remodeling of the extracellular matrix [103,135,137,212,215,218] and growth behavior [15,78,108,131,133,135,212,218,237]. Cellular changes were detected in embryonic stem cells, mesenchymal stem cells from different origins, epidermal stem cells, adipose stem cells, hematopoietic progenitor cells, cardiovascular progenitors, NK cells, retinal pigment epithelium cells, aortic smooth muscle cells, vascular smooth muscle cells, osteoblasts, lymphocytes, endothelial cells, macrophages, chondrocytes, thyrocytes, and others.

Differences in the metabolomic profile in space-flown oligodendrocytes [94] and an altered metabolism in s-µ*g*-exposed epidermal stem cells have been reported [79]. Furthermore, several cellular signaling pathways were influenced by spaceflight. Prominent examples are the STAT3 [103,107], NFκB [103], PI3K/AKT [103], HIF1α [103], VEGF [126,129] and endothelin pathways, among others [103,126].

Taken together, these changes included modifications of the cytoskeleton, which was subject to substantial reorganization affecting both cell shape and internal organization (e.g., accumulation of intermediate filaments around the nucleus, stress fibers), and alterations in the ECM, influencing cell–cell and cell–matrix interactions [20]. Additionally, changes in cell adhesion and focal adhesion [192] affected how cells attach to the culture flasks and interact with their environment, while their migration behavior was also altered under µ*g* conditions. 

The findings summarized in this review also underscored the combination of omics studies and artificial intelligence (AI) to analyze the complex biological responses to µ*g*, to advance translational regenerative medicine, and to ensure the astronauts’ health during long-term space missions.

In addition, µ*g* exposure led to various cellular adaptations. Stem and progenitor cells exhibited modified differentiation patterns, potentially impacting tissue regeneration and homeostasis (Table 3). Cell division rates and overall growth patterns were affected, with implications for tissue maintenance and repair. Furthermore, µ*g* influenced cell survival and apoptosis mechanisms. One of the most promising µ*g* effects is its impact on three-dimensional cell growth. µ*g* conditions facilitated the development of more complex, tissue-like structures such as 3D organoids and multicellular spheroids, thus enhancing their potential for tissue engineering and biofabrication applications [275]. The absence of sedimentation and convection in space creates unique opportunities for bioengineering.

This review highlights that µg influences various omics levels in human cells, including genomics, transcriptomics, epigenomics, metabolomics, and proteomics. These changes were observed across the different cell types discussed in this review. µ*g* exposure changed gene expression patterns, potentially affecting cellular function and adaptation mechanisms [96]. RNA regulation was highly adaptable and transient, showing rapid changes in response to µ*g* conditions. There were clear differences between short- (parabolic flight) and long-term r-µ*g* investigations (spaceflight) regarding the gene expression pattern [276]. Alterations in epigenetic markers and regulatory mechanisms suggest long-term adaptations to µ*g*. While the protein secretion and hormone/cytokine expression changes occurred more slowly than transcriptomic changes, they often correlated with meaningful functional alterations in the cells [133,135,276]. The current research indicates that µ*g* had notable effects on cardiac progenitor cells and human-induced pluripotent stem cell-derived cardiomyocytes, resulting in alterations in the expression patterns of genes associated with cardiac function and metabolism (Section 4.4). Moreover, µ*g* influenced immune cells such as T cells and macrophages, suppressing critical genes and pathways involved in immune responses (Table 5). Additionally, crucial for joint health, chondrocytes displayed modified gene expression and cytoskeletal structures under µ*g* conditions, potentially contributing to degenerative joint diseases [215,216,217]. In summary, µ*g* induced significant changes in cellular behavior, gene and protein expression, as well as metabolic processes, impacting various systems within the human body.

One important question to be answered is whether we can conclude from the available literature that the results obtained from s-µ*g* studies coincide with the findings resulting from studies on the ISS or in space, in general. This review revealed similarities between the effects observed in r-µ*g* on the ISS and s-µ*g* conditions [133,135,172,190,276]. For example, cartilage tissue engineering was possible in both space and on Earth using the RWV bioreactor or the RPM [212,218,277,278]. Among others, changes in the ECM, cytokines, growth, and survival were detected in space and under s-µ*g* conditions.

Ground-based facilities were used to prepare for space experiments studying endothelial cells or thyroid cancer cells in space, yielding comparable results [18,26,133,135,244,245]. Similar morphological and functional changes were observed in both r-μ*g* and s-μ*g* conditions [131,133,135]. Omics studies from both environments revealed comparable alterations in the gene expression patterns, protein profiles, and the secretome [131,135]. 

Moreover, this review also emphasizes essential considerations. Different simulation methods may have varying effects on cells, necessitating careful interpretation of the results [53,54] and further comparative studies. Some simulation techniques may introduce confounding factors like shear and hydrodynamic stress [49,52]. The importance of an in-flight 1*g* reference centrifuge is stressed to distinguish µ*g* effects from other spaceflight factors like cosmic radiation. Validation of ground-based results with r-µ*g* experiments is important to ensure accuracy and reliability. The findings from these studies have significant implications for various fields. Understanding cellular responses to µ*g* in space exploration is key to maintaining astronaut health during long-duration missions. In regenerative medicine, µ*g*-induced three-dimensional growth enhancement offers new possibilities for tissue engineering and organoid development [279] as well as pharmacology and toxicology. For medicine on the ground, results obtained from µ*g* research may lead to new treatment strategies for Earth-based medical conditions. In the field of biotechnology and translational regenerative medicine, the unique conditions of µ*g* provide opportunities for innovative bioengineering and biofabrication techniques [279]. 

Taken together, while there is a general trend between r-µ*g* (ISS, spaceflight) and s-µ*g* findings, this review supports the urgent need for continued research and validation to fully understand the complex effects of µ*g* on cellular function and adaptation.

## 6. Conclusions

This review summarizes and discusses the current knowledge about omics investigations of stem cells, benign specialized cells, and humans and animals exposed to altered gravity conditions on Earth and in space. 

The space age has just begun with the exploration of other planets and space tourism. We must know that space stressors like µ*g*, radiation, isolation, and psychological problems can impact astronauts, cosmonauts, taikonauts, and space tourists’ health. The successful development of future lunar and Martian settlements and extended space voyages requires rigorous organization and the identification of all potential hazards to humans and animals, coupled with robust countermeasures. Current knowledge indicates that human organisms, animals, bacteria, and plants will undergo significant changes at the cellular level [275]. Novel strategies to fight these spaceflight-associated diseases must be developed. Over the past 25 years, numerous articles have explored the impact of microgravity on the cytoskeleton, ECM, adhesion, migration, differentiation, proliferation, survival, apoptosis, autophagy, growth, etc. [13,25,136,261,280,281]. Various countermeasures have been developed that are important to be applied during spaceflights, for example, against muscle and bone loss [282,283].

Omics studies can contribute to expanding the current knowledge in this field, which requires a multifaceted approach—RNA sequencing (RNA-seq) for comprehensive transcriptome analysis, proteomics using mass spectrometry, and bioinformatics tools for in silico data evaluation are essential [96].

The generation and AI-based integration of transcriptome, proteome, and epigenome data enables the construction of complex interaction networks. It offers a new framework for understanding the molecular basis of μ*g*-induced physiology and its derivation to the pathophysiological conditions (Figure 3).

Explainable AI (XAI) models are invaluable in space research, serving as decision support systems to identify key drivers within complex molecular systems. Diseases like cancer can be supported by AI models, as shown in medical research in the last few years [284,285,286]. Due to the increasing demand for AI that is not directly interpretable, comprehensible, and trustworthy, it is important to find a good trade-off between the performance of a model and its transparency [287]. Moreover, understanding the decision-making process within an AI model can lead to correcting its shortcomings and help to (i) correct biases in the training dataset, (ii) increase robustness by highlighting potential confounds, and (iii) guarantee that only meaningful variables derive the output [288]. AI is conquering space research because it is a powerful method that helps researchers find correlations beyond humans’ cognitive reach. Many research studies focus on performance metrics and outcomes [289]. The Space Omics and Medical Atlas (SOMA) and the international astronaut biobank [286] are important new tools to support precision space medicine. International, interdisciplinary collaboration is essential to ensure the convergence of omics and artificial intelligence in space research.

## Figures and Tables

**Table 1:** Omics studies on stem cells under microgravity conditions.

Cell Line	Omics	µ*g*	Device/Platforms	Refs.
Embryonic stem cells (ESCs)	Proliferation: mixed results (PCNA expression), decreased adhesion rates, increased apoptosis rates, inhibited differentiation into mesodermal (FoxA2, Sox17), cardiomyocyte lineages (WNT1).	s-µ*g*, r-µ*g*	3D clinostat, 7 d Cargo spacecraft, 15 d Rotary bioreactor, 6 d NASA STS-131, 15 d	[68,69,71,72]
Mesenchymal stem cells (MSCs)	Decreased oxidative phosphorylation (PGC-1α), mtDNA copy number, mitochondrial mass, and oxygen consumption rate. Decreased Sirt1 expression, increased YAP expression, mitochondrial dysfunction, and senescence. Downregulated expression of Oct-4, SOX2, NANOG. Initial upregulated expression of p16, p19, p21, p53 but after 48 h p19, p21, p53 expressions were downregulated. Upregulated expression of HSP60, HSP70. Increased secretion of neural differentiation (neurotrophins: TH, CHAT, MAP-2, NGF, BDNF, CNTF). Disrupted MAPK signaling (AKT, ERK1/2) pathway.	s-µ*g*	Clinostat, 72 h Clinostat, 72 h Clinostat, 48 h Clinostat, 72 h Clinorotation, 3 d	[73,74,75,76,77]
Epidermal stem cells (EpSCs)	Increased proliferation (Ki67), decreased differentiation (variolatin markers). Lipid metabolomics: upregulation of phosphatidylethanolamine (PE), downregulation of phosphatidylcholine (PCs) and phosphatidylserine (PSs). Reduction in ceramides, and elevation of sphingomyelin observed.	s-µ*g*	RCCS, 15 d RCCS, 3 d	[78,79]
Cancer stem cells (CSCs)	Downregulated stemness markers (CD44, CD133, ALDH, Nanog, Oct-4). Increased apoptosis and CSC proliferation (CD133+).	s-µ*g*	RPM, 48 h NASA developed a hydrodynamic focusing bioreactor (HFB), 7 d	[80,81]
Hematopoietic stem cells (HSCs)	Inhibited migration potential (SDF-1a) in bone marrow CD34+, disordered cytoskeleton (decreased F-actin formation and microfilament reorganization), decrease in activity in NK cells, arrest in cell cycle (G0/G1 phase), prolonged S phase, reduced cyclin-A expression, downregulation of SCF pathway, suppressed metabolism (ether lipid metabolism, unsaturated fatty acid biosynthesis, glycolipid metabolism). Reduction in immune cell populations NK cells, B cells, erythrocyte precursors, increased T cells and neutrophils, increased macrophages, granular leukocytes, monocytes.	s-µ*g* r-µ*g*	RWV, 18d, clinostat, 72 h SJ-10 satellites, Tianzhou-1 cargo ship, and RWV, 12 d. Spaceflight, Shenzhou 9 d or 10 d, 13 d and 15 d, Hindlimb unloaded mice, 28 d. HU mice model, 28 d.	[82,83,84,87,88]

**Table 3:** Omics studies on cardiac cells under microgravity conditions.

Cell Line/Tissue/Sample Type	Omics	µ*g*	Device/Platforms	Refs.
Human-induced pluripotent stem cell-derived cardiomyocytes	Transcriptomics—3008 DEGs between ground and flight samples 1049 DEGs between post-flight and ground samples Upregulation of hypertrophy-associated genes (MEF2D, cardiac troponin T, troponin 11) in flight samples but not in post-flight samples	r-µ*g*	ISS, SpaceX Falcon 9 rocket, 5.5 weeks	[119]
Human aortic smooth muscle cells	Transcriptomics—4422 DEGs between ground and flight samples Markers of the three main phenotypic profiles were downregulated, including smooth muscle alpha-actin (αSMA), matrix metalloproteinases (MMPs), and bone morphogenic proteins (BMPs).	r-µ*g*	ISS, SpaceX Falcon 9 rocket, 3 d	[103]
Human- induced pluripotent stem cell-derived cardiac progenitors (CPCs)	Transcriptomics—470 DEGs between ground and flight samples Upregulation of genes related to cardiac function (THBD, GADL1), contraction (MYL2, TNNI3, SCN5A, RYR2), conduction (ATP2B4, PRKACA, ANK2), proliferation and cardiac differentiation (CCBE1, CCND2, IGFBP5, BDKRB2) Downregulation of genes related to ECM regulation (ITIH5, COL4A4, PTPR21, COL26A1, HPX) and focal adhesion (ITGA11, PROCR, ANXA1, LIMA1)	r-µ*g*	ISS, SpaceX CRS 20 mission, 3 weeks	[29]
Human-induced pluripotent stem cell-derived cardiomyocytes	Supernatant metabolomics—13 significantly different metabolites between ground and flight samples Higher content of thiamine, 2-hydroxy-2-methylbutanedioic acid, 4-aminoindole. Transcriptomics—downregulation in ACO2, IDH1, IDH2, OGDHL, SUCLA2, and SDHD.	r-µ*g*	CSS, Shenzhou-13 capsule, 6 d	[124]
Neonatal and adult human-induced pluripotent stem cell-derived CPCs	Transcriptomics—10,565 upregulated genes in adult CPCs (SOX2, KLF4, MESP1, NKX2.5, GPR22, GPR142, GPR6) 13,484 upregulated genes in neonatal CPCs (SOX2, KLF4, CXCR4, MESP1, NKX2.5, GPR22, GPR142, GPR6)	r-µ*g*	ISS, SpaceX CRS-11 mission, 30 d	[114]
AC16 cells (human cardiomyocyte cell line) treated 10 days before launch (L-10) and 3 days after return (R+3) astronaut-derived exosomes for 24 h	Transcriptomics—186 DEGs between L-10 and R+3 groups (enhancement in SMAD4, SUZ12, IRF8, GATA2, and VDR). Epigenomics—H3K27me3 enrichment in the VDR promoter in an EZH2-dependent manner	r-µ*g*	Astronaut-derived exosomes, 14 d	[125]
Neonatal and adult human-induced pluripotent stem cell-derived CPCs	Transcriptomics—adult CPCs (upregulation in YAP1, ATM, TERT; downregulation in NES) Neonatal CPCs upregulation related to embryonic stem cell self-renewal (*POU5F1*, *NANOG*, *SOX2*), DNA repair genes (*RAD23*, *RAD50*), mesodermal specification (*FOXC1*, *MESP1*), and early cardiogenesis (*GATA4*, *KIT*, *PDGFRA*, *ISL1*, *KDR*)	r-µ*g*	ISS, SpaceX CRS-11 mission, 12 d	[115]
Human-induced pluripotent stem cell-derived cardiomyocytes	Transcriptomics—825 DEGs between ground control and simulated microgravity samples. Proteomics—56 down- and 123 upregulated proteins between control and simulated microgravity samples.	s-µ*g*	Slide-flask 2D clinostat	[104]

**Table 4:** Omics studies on endothelial cells under microgravity conditions.

Cell Line	Omics	µ*g*	Device/Platforms	Ref.
Endothelial cells EA.hy926 cells	Caspase-3, Bax, and Bcl-2 proteins were increased	s-µ*g*	3D clinostat, 10 d	[126]
Endothelial cells EA.hy926 cells	IL-8 (interleukin-8) and VWF (von Willebrand factor) genes were most prominently affected.	s-µ*g*	RPM, 5 d and 7 d	[18]
Endothelial cells EA.hy926	VEGF, NGAL, IL-6, IL-8, MCP-1, VCAM-1, ICAM-1, fibronectin, and RANTES were shown to be affected	s-µ*g*	RPM, 35 d	[133]
Endothelial cells EA.hy926 ECs	1023 significantly modulated genes: thioredoxin-interacting protein was the most upregulated (33-fold), and heat-shock proteins 70 and 90 were the most downregulated (5.6-fold).	r-µ*g*	Parabolic flight	[137]
Endothelial cells EA.hy926	Enhanced collagen and laminin were detected	r-µ*g*	ISS, 12 d	[135]
Endothelial cells EA.hy926 and human microvascular ECs (HMVEC)	Proteome analysis revealed that 3D structures of HMVEC showed a transient augmentation of ribosomal proteins.	s-µ*g*	RPM, 5 d and 7 d	[131]
Human umbilical vein endothelial cells (HUVECs)	Increase in the inducible nitric oxide synthase (iNOS) expression	s-µ*g*	Clinostat, 24 h	[127]
Human umbilical vein endothelial cells (HUVECs)	MicroRNA: hsa-mir-496, hsa-mir-151a, hsa-miR-296-3p, hsa-mir-148a, hsa-miR-365b-5p, hsa-miR-3687, hsa-mir-454, hsa-miR-155-5p, and hsa-miR-145-5p differentially regulated gene pathways	s-µ*g*	Clinostat, 2 h	[129]
Human umbilical vein endothelial cells (HUVECs)	Decrease in the secretion of key pro-inflammatory cytokines (IL-1α and IL-8) and pro-angiogenic factor bFGF.	s-µ*g*	RPM, 96 h	[130]
HUVECs	Change in the expression of 177 genes. Clusters of cell response to external stimuli and regulation of cell motility and proliferation among the overexpressed genes.	s-µ*g*	RPM, 24 h	[132]
Human dermal microvascular endothelial cells (HMVEC-dBL)	Transcriptomic analysis revealed suppression of Lbp, MyD88, and MD-2.	r-µ*g*	ISS, 10 d	[139]
Human dermal microvascular endothelial cells (HMVEC-dBL)	Several markers linked to energy deficiency, and amino acids such as tryptophan, creatinine, dopamine, and glycine, and cofactors such as lactate and pyruvate were altered	r-µ*g*	ISS, 10 d	[134]
Human microvascular endothelial cells (HMEC-1)	DNA repair mechanisms were found to be activated in microgravity, and the transcriptomics analysis showed the opposite effects of microgravity and space radiation.	r-µ*g*	ISS, 5 d	[136]
Human pulmonary microvascular endothelial cells (HPMECs)	Downregulation of PI3K/AKT pathway, increased expression of NF-kB	s-µ*g*	Clinostat (MG-3), 72 h	[128]
Human aortic smooth muscle cells (HASMCs)	4422 genes altered, cells downregulate markers of the contractile, synthetic, and osteogenic phenotypes like smooth muscle alpha-actin (αSMA), matrix metalloproteinases (MMPs), and bone morphogenic proteins (BMPs).	r-µ*g*	ISS, 3 d	[103]

**Table 5:** Omics studies on the hematological system under microgravity conditions.

Cell Line	Omics	µ*g*	Device/Platforms	Refs.
PBMCs	Immune cell transcriptional response alteration to ConA/anti-CD28 stimulation Decrease the mRNA expression of IL-2Rα, TNF-α, CD69, CLL4 and IFN-γ	s-µ*g*	RWV, 18 h	[148]
	Shorter telomeres, lower telomerase activity, and increased chromosomal abnormalities	r-µ*g*	Space shuttle mission, one one-year mission crewmember, and ten six-month mission crewmembers	[155,156]
	Inhibits LPS-dependent Jun-N-terminal kinase activation triggered by LPs	r-µ*g*	Soyuz spacecraft, 5 to 6 min of microgravity	[157]
	Change in immune-related pathways Changes in telomere length	r-µ*g*	ISS, one year	[123]
	Increase in the expression of beta 2-integrins IL-6 increasing significantly	s-µ*g*	HDTBR head-down bed rest, 120 d	[161]
	Significant decrease in IL-2 (interleukin-2), interferon-	s-µ*g*	HDTBR head-down bed rest, 21 d	[162]
	Downregulation of T cell activation genes (DAG kinase, human serine/threonine kinase and tyrosine kinase) Upregulation of most of the 176 genes	s-µ*g*	Microgravity analog culture system (MAGS), 24 h and 72 h	[177]
	Elevated mitochondrial dysfunction and increased oxidative damage, inflammation, and DNA damage	r-µ*g*	Space shuttle mission, 33 d and 37 d	[154]
	Downregulation of genes *HSP27*, *BAG-1*, *XRCC-1*, *HPS90AB1*, *GPX1*, *HHR23A*, *FAP48*, and upregulation of *C-FOS*	r-µ*g*	Space shuttle mission, 10 d and 13 d	[151]
Leukocytes	Elevated levels of inflammatory markers such as TNF-α, IFN-α and IFN-γ, IL-1, IL-4, IL-10, IL-12, IL-6 Alteration in cytokine production profiles, particularly IFN-γ, IL-7, and IL-10	r-µ*g*	Space shuttle mission, 10 d and 15 d	[150]
	Reduced leukocyte adhesion molecule CD62L expression Activation of signaling pathways related to immune response	s-µ*g*	HDTBR head-down bed rest, 5 d of HDTBR or daily 30 min of centrifugation or 6 × 5 min of centrifugation	[160]
T cell (CD8 and CD4)	Inhibiting the PSTAT5 responses Suppression key aspects of CD4T, NK cell and CD8 T cell including CD25, CD69 and JAK/STAT signaling	s-µ*g*	RWV, 18 h	[148]
	Significant variation in cytokine levels Changes in DNA methylation, Epigenetic discordance in the promoters of *NOTCH3* and *SLC1A5*/*ASCT2*	r-µ*g*	ISS, one year	[123]
T lymphocytes	NFAT binding to DNA was impaired owing to the downregulation of AP-1	s-µ*g*	Rotary cell culture system, 2.5 h and 16 h	[179]
T help 1	Reduced cytokines, IL-2, IFN-γ, and TNF-α	s-µ*g*	HDTBR head-down bed rest, 21 d	[162]
CD4 T cell	Slight impaired DAG signaling downstream of the TCR	s-µ*g*	RWV, 5–90 min	[169]
	Decreased IgM and IgG antibody production	s-µ*g*	RCCS, 1 d, 2 d, 6 d and 12 d	[170]
Lymphocytes	Rise in apoptotic markers such as DNA fragmentation and cleaved-poly (ADP-ribose) polymerase protein expression increase in P53 and calpain mRNA expression	r-µ*g*	BIO-4 missions, 3 h, 24 h, and 48 h	[172]
Neutrophils	Increased oxidative stress Increased of neutrophils number oxidative burst capacity	r-µ*g*	Space shuttle mission, 5 d to 11 d	[153]
	Increase in myeloperoxidase expression	s-µ*g*	RCCS, 20 h	[163]
Monocytes	Reduction in IL-6 production Reduction in the expression of CD62L and HLA-DR, TNF-α, and IL-10	r-µ*g*	Space shuttle mission, 13–16 d	[184]
	Significantly reduction in phagocytic by expression changes in CD32 and CD64	r-µ*g*	Space shuttle mission, 5–11 d	[153]
Dendritic cells (DCs)	Downregulation of genes encoding CD40, CD80, CD83, and NF-κB	r-µ*g*	Eneide spaceflight mission, 24 h	[158]
B cells: CD19 and lymphocyte depleted (LD)	112 differentially expressed genes	r-µ*g*	ISS, 6 d	[123]
Primary human CD41	Reduced abundance of CD3 and ZAP70, IL-2R	s-µ*g*	2D clinorotation, 5–60 min	[167]
NK cells	Noticeable decrease in interferon IFN-γ and perforin production There is a slight decrease in granzyme-ß production Reduction in receptors NKG2A, NKG2D	s-µ*g*	2-DRWV, 12 h, 24 h, 48 h and 72 h	[171]
M1 macrophages	Decrease in ICAM-1	r-µ*g*	ISS, 11 d and 30 d	[173]
	Decrease in binding sites for the epigenetic marker H3K4me3 Altered transcriptional activity	s-µ*g*	Parabolic flight, 20 s	[175]
M2 macrophages	8 metabolites revealed significantly different quantities	r-µ*g*	Cell box primary human macrophage experiment, 3 h, and 6 min	[180]
M1 and M2 macrophages	Increase in MCP-1 and IL-1ra Increase in chemokine ligand 4/macrophage inhibitory protein CXCL5	r-µ*g*	ISS, 30 d	[183]
Human lymphoblastoid cells	Epigenomic alterations and changes in the methylome 60% of DMRs showed reduced methylation levels 370 transcripts were identified with altered expression	s-µ*g*	HARV rotary cell culture system, 48 h	[176]
Primary T cells	Suppression of the expression of miR-21	r-µ*g*	ISS	[189]
Primary macrophages	Protein degradation Glucogenic and ketogenic amino acids were further degraded to produce glucose and ketoleucine	r-µ*g*	Sounding rocket, 11 d	[195]
B lymphoblast HMy2.CIR	Enhanced production of ROS induction of ROS-sensitive ERK/MKP-1/caspase-3 activation	s-µ*g*	RWV, 30 min	[196]

**Table 6:** Omics studies on the musculoskeletal system under microgravity conditions.

Cell Line/Tissue/Sample Type	Omics	µ*g*	Device/Platforms	Refs.
Human meniscus fibrochondrocytes	Upregulation of key osteoarthritis markers *COL10A1*, *MMP13*, and *SPP1* Downregulating chondrogenic markers ACAN, *COL1A2*, *COL2A1*, and *SOX9*	s-µ*g*	RCCS Bioreactor, 7 d	[214]
Human chondrocytes	Upregulation of inflammatory markers *IL6* and *CXCL8* Downregulation of growth factors EGF, VEGF, and FGF17 Alteration in PI3K-Akt signaling pathway and Stress response pathways	r-µ*g*	DLR parabolic flight campaign	[217]
Human chondrocytes	Upregulation of BMP-2 and SOX9 genes mitigating microgravity-induced cytoskeletal alteration	r-µ*g*	Airbus A300 Zero-G parabolic flight	[216]
Human chondrocytes	Increased TGF-β1 protein production Upregulation F-actin and ACTB	s-µ*g*	RPM, 30 m, 2 h, 4 h, 16 h, 24 h	[215]
Human chondrocyte cell line C28/I2	Significant changes in the regulation of *IL6*, *ACTB*, *TUBB*, *VIM*, *COL1A1*, *COL10A1*, *MMP1*, *MMP3*, *MMP13*, *ITGB1*, *LAMA1*, *RUNX3*, *SOX9*, and *CASP3* gene expression	s-µ*g*	RCCS-2D	[218]
Meniscus fibrochondrocytes	Significant upregulation of *CLEC2B* and *RMGA* Sex-specific gene expression changes: upregulated *KTI12*, *HSPE-MOB4*, *WNT7B* and *WNT9A* and *HSPA1A* in females and *HSPA1B* in males	r-µ*g*	Falcon 20 shuttle, 11 parabolas	[36]
Blood	Secretomics—no change in neuromuscular secretome under bed rest with or without centrifugation	s-µ*g*	6° head-down tilt bed rest, 60 d	[100]
Combination of over 20 datasets from different cell types, tissues, and blood and urine samples	Transcriptomics, metabolomics-altered mitochondrial function and mitochondrial stress	r-µ*g*	space flight, various lengths	[154]
Blood	Secretomics—markers for metabolism and early stages of muscle regeneration elevated in flight, decreased after landing	r-µ*g*	space flight on the ISS, 340 d	[182]
Muscle biopsies	Proteomics—downregulation of proteins involved in focal adhesions, fiber–matrix interaction, and insulin receptor stabilization, reverted by reloading	s-µ*g* r-µ*g*	6° head-down tilt bed rest, 10 d; space flight on the ISS, 6 months	[219]
Muscle biopsies	Proteomics—strong decrease in mitochondrial and oxidative phosphorylation-related proteins, quick reversion after landing	r-µ*g*	space flight on the ISS, 6 months	[220]
Muscle biopsies	Proteomics—bed rest alone, bed rest with exercise, and bed rest with exercise complemented by testosterone-induced distinct protein expression and phosphorylation patterns (proteins related to muscle contraction vs. cellular organization in muscle vs. mesenchymal migration)	s-µ*g*	head-down tilt bed rest, 70 d	[221]
Muscle biopsies	Transcriptomics—exercise prevents the sensitization of dorsal root ganglion neurons	s-µ*g*	6° head-down tilt bed rest, 5 weeks	[92]
Muscle biopsies	Proteomics—Exercise prevented an increase in myosin heavy chain type I and a decrease in type II in vastus lateralis and calf soleus	s-µ*g*	bed rest, 55 d	[222]
Muscle biopsies	Proteomic data from [PMID: 20957755] as a base for in silico miRNA expression prediction—let-7a-5p, miR-125b-5p, miR-1-3p, miR-125b-5p, miR-1-3p as well as miR-95-5p possibly involved in observed protein regulations	s-µ*g*	bed rest, 55 d	[223]
Muscle biopsies	Transcriptomics, proteomics—Resistive training with vibrations reduced the number of differentially expressed genes from 235 to 51 compared to bed rest alone. Mostly, metabolic pathways were affected	s-µ*g*	6° head-down tilt bed rest, 60 d	[224]
Human blood-derived stem cells	Proteomics, epigenomics -µ*g* accelerated osteogenic differentiation, decreased Sox2, Oct3/4, Nanog, and E-cadherin protein expression, mediated Otx2, Snail, GATA4, and Sox17 expression. Involvement of H3K4me3, H3K27me2/3, H3K79me2/3, and H3K9me2/3	r-µ*g*	Space flight on the ISS, 48 and 72 h	[225]
Human bone marrow mesenchymal stem cells	Transcriptomics—2 d: downregulation of cell cycle, mitosis, cell division, or nuclear division 7 d: upregulation of genes involved in the PPAR signaling pathway and suppression of genes from calcium signaling pathway, pancreatic secretion, or GnRH signaling pathways 14 d: most prominently enriched pathways related to tumorigenesis	s-µ*g*	RPM, 2 d, 7 d, and 14 d	[226]
Osteocommitted human mesenchymal stromal cells	Transcriptomics—regulation of *COL11A1*, *CTNNB1*, *HAS1*, *ITGA3*, *ITGB1*, *LAMA3*, *MMP1*, *MMP11*, and *TNC*	s-µ*g*	RPM, 10 d	[227]
Human osteoblast-like MG-63	Transcriptomics—TLN1, SPTBN1, COTL1, and WASF2 significantly regulated in µ*g* vs. control	s-µ*g*	diamagnetic levitation	[228]
Human bone marrow stem cells	Transcriptomics—*PPRy*, *SOX9*, *MEK/ERK*, *LIP*, *Msx2*, *RB1*, *S100a16*, *Mir29A*, *SFRP-4*, *Mir31*, *MirLetA2*, *RUNX2*, *COL1A*, *BGLAP*, *RANKL*, *ALP*, *CYP27A*, and *TAZ* have a central role in mechanosensing	r-µ*g*	Soyuz, ISS, 14 d	[229]
Human bone marrow stem cells	Transcriptomics—GSN-AS1, EPB41L4A-AS1, TP53TG1, MIR155HG, RNF185-AS1, and SNHG12 as hub lncRNAs in a coding–non-coding gene co-expression network	r-µ*g*	Soyuz, ISS, 14 d	[230]
A mixture of different cells and tissues from different species	Transcriptomics—*IL6*, *MAPK3*, *JUN*, *CXCL8*, *IL18*, *FGF2*, *IGF1*, *PTGS2*, *CCL2*, *FOS*, and *ICAM1* as hubs in a µ*g*-sensitive gene regulation network	s-µ*g*, r-µ*g*	RPM, RWV, RCCS, spaceflight	[231]
Human bone marrow stromal cells	Proteomics—481 differentially abundant protein groups (DAPGs), with proliferation, differentiation, adhesion, signaling, death, carbohydrates, lipids, proteins, transport, and nucleic acids as enriched biological processes	s-µ*g*	RPM, 8 d and 28 d	[232]
Human primary osteoblast	Proteomics, metabolomics—>50% of RPM-sensitive genes belonged to the mitochondrion and mitochondrial processes. Significant reduction in glycolysis, Krebs cycle, PPP, the glycerol-phosphate shuttle, as well the malate-aspartate shuttle on the RPM	s-µ*g*	RPM, 110 h	[233]

**Table 7:** Omics studies on the endocrine system under microgravity conditions.

Cell Line/Tissue/Sample Type	Omics	µ*g*	Device/Platforms	Refs.
Normal prostate	Prostatic acid phosphatase (PAP) decreased cytokeratins, vimentin, and TGF-beta2 receptors and ligands were preserved	s-µ*g*	RWV, 28 d	[239]
Normal human HTU-5 thyroid cells	Moderate signs of apoptosis, caspase-3 activation, elevation of Fas protein 85 kDa apoptosis-related cleavage fragments resulting from enhanced poly(ADP-ribose) polymerase activity	s-µ*g*	3D clinostat, 24 h	[242]
FRTL5 thyroid cells	No response to TSH Changes in morphology and shedding of the TSH receptor elevation of sphingomyelin-synthase and Bax proteins	r-µ*g*	TEXUS-44 mission	[243]
Nthy-ori 3-1 thyroid cells	Differences between benign Nthy-ori 3-1 and malignant FTC-133 cells: expression of *NGAL*, *VEGFA*, *SPP1*, *IL6,* and *IL17* mRNAs and the secretion of VEGFA, IL-17, and IL-6 proteins.	s-µ*g*	RPM, 7 d and 14 d	[244]
Nthy-ori 3-1 thyroid cells	4 h: upregulation of *IL6*, *CXCL8* and *CCL2* mRNAs in AD cells vs. 1*g* 24 h: downregulation of *IL6*, *CXCL8*, *FN1*, *ITGB1*, *LAMA1*, *CCL2*, and *TLN1* mRNA in MCS vs. 1*g* 72 h: increase in secretion of IL-6, IL-8, and TIMP-1	s-µ*g*	RPM, 4 h, 24 h, and 72 h	[245]
Nthy-ori 3-1 thyroid cells	No influence of DEX on spheroid formation s-µ*g* induces upregulation of the anti-adhesion protein mucin-1 8-fold increase in fibronectin in DEX-treated Nthy-ori 3-1 cells	s-µ*g*	RPM, 72 h DEX concentrations (0, 10, 100, 1000 nM)	[246]
Pancreatic β-cells	MIN6 spheroids co-expressed insulin and C-peptide Insulin-2, glucokinase, SETD1A, and Kir6.2 genes were more highly expressed in the spheroids generated in the 3D culture of MIN6 cells than in the MIN6-2D cells There is no significant difference between the expression levels of the insulin1 and glucose transporter type genes in the spheroids and MIN6-2D cells. Insulin secretion in response to the elevation of the glucose concentration in spheroids was higher vs. MIN6-2D cells	s-µ*g*	3D clinostat, 4 d	[247]
PHHI-derived islet cells	3D pseudo-islet formation in s-µ*g* In s-µ*g:* reactivation of insulin, glucagon, somatostatin, and GAD expression in PHHI-derived cells that had previously stopped their expression	s-µ*g*	HARV 2 d, 5 d, 7 d, and 20 d	[248]

**Table 9:** Omics studies on the skin under microgravity conditions.

Cell Line	Omics	µ*g*	Device/Platforms	Refs.
Human keratinocytes (HaCaT)	Transcriptomics (increased expression of MMP1, MMP2, MMP3, MMP9, SNAIL1, SNAIL2, ZEB2) Translatomics (increased Snail1, alpha-SMA, vimentin, fascin) Secretomics (increased MMP2 and MMP9 activity)	s-µ*g*	RPM, 6 h, 24 h and 60 h	[265]
Primary cultures of human dermal fibroblasts	Proteomics (increased protein carbonylation by oxidative stress) Secretomics (decreased MMP-9 activity) Metabolomics (increased apoptosis on RPM)	s-µ*g*	3D clinostat, 24 h and 72 h	[266]
Human epidermal keratinocytes HaCaT	Secretomics (increased collagen production) Transcriptomics (increased *COL1A1* expression) Metabolomics (no impaired cell viability)	s-µ*g*	3D clinostat, 24 h, 48 h and 72 h	[260]
Primary human dermal fibroblasts	Secretomics (decreased collagen production in space)	r-µ*g*	Spacelab D2-mission, 4 h, 7 h, 10 h, and 20 h	[268]
Juvenile normal human dermal fibroblasts	Transcriptomics (*FN1* upregulation, *ACAN* downregulation in MCS) Translatomics (fibronectin upregulation in MCS) Secretomics (decreased MCP-1, increased JNK1 in MCS) Metabolomics (multiple pathway alterations, cytoskeleton disruption in s-µ*g*)	s-µ*g*	RPM, 72 h	[270]
Primary normal human dermal fibroblasts	Translatomics (decreased procollagen type III, increased fibronectin under s-µ*g* and cortisone) Secretomics (decreased IL-6 secretion under s-µ*g* and cortisone) Metabolomics (actin stress fiber reduction under s-µ*g* and cortisone, decreased number of focal adhesions)	s-µ*g*, hyper-*g*	RPM, 24 h Large Diameter Centrifuge, hydrocortisone 1 µM	[271]
Primary skin tissue from C57BL/6J *Mus musculus* mice	Metabolomics, secretomics (relative increase in type III collagen-rich fibers under r-µ*g*, “looseness” of ECM, changes in ECM distribution, increased mast cell granule secretion)	r-µ*g*	ISS, 21–24 d	[272]
Human dermal microvascular endothelial cells (HDMEC)	Metabolomics (cell division rate not impaired, ROS upregulation), translatomics (HSP70 upregulation)	s-µ*g*	RWW, 4 d and 10 d	[273]
